# Dementia with Lewy Bodies: Molecular Pathology in the Frontal Cortex in Typical and Rapidly Progressive Forms

**DOI:** 10.3389/fneur.2017.00089

**Published:** 2017-03-13

**Authors:** Paula Garcia-Esparcia, Irene López-González, Oriol Grau-Rivera, María Francisca García-Garrido, Anusha Konetti, Franc Llorens, Saima Zafar, Margarita Carmona, José Antonio del Rio, Inga Zerr, Ellen Gelpi, Isidro Ferrer

**Affiliations:** ^1^Institute of Neuropathology, Service of Pathologic Anatomy, IDIBELL-Hospital Universitari de Bellvitge, Hospitalet de Llobregat, Barcelona, Spain; ^2^CIBERNED, Network Centre for Biomedical Research of Neurodegenerative Diseases, Institute Carlos III, Madrid, Spain; ^3^Neurological Tissue Bank of the Biobanc-Hospital Clínic-Institut d’Investigacions Biomèdiques August Pi I Sunyer (IDIBAPS), Barcelona, Spain; ^4^Department of Neurology, Clinical Dementia Center, University Medical School, Georg-August University, German Center for Neurodegenerative Diseases (DZNE), Göttingen, Germany; ^5^Molecular and Cellular Neurobiotechnology, Department of Cell Biology, Institute of Bioengineering of Catalonia (IBEC), Parc Científic de Barcelona, University of Barcelona, Barcelona, Spain; ^6^Department of Pathology and Experimental Therapeutics, L’Hospitalet de Llobregat, University of Barcelona, Barcelona, Spain

**Keywords:** dementia with Lewy bodies, Alzheimer’s disease, α-synuclein, mitochondria, protein synthesis, inflammation, β-amyloid, olfactory receptors

## Abstract

**Objectives:**

The goal of this study was to assess mitochondrial function, energy, and purine metabolism, protein synthesis machinery from the nucleolus to the ribosome, inflammation, and expression of newly identified ectopic olfactory receptors (ORs) and taste receptors (TASRs) in the frontal cortex of typical cases of dementia with Lewy bodies (DLB) and cases with rapid clinical course (rpDLB: 2 years or less) compared with middle-aged non-affected individuals, in order to learn about the biochemical abnormalities underlying Lewy body pathology.

**Methods:**

Real-time quantitative PCR, mitochondrial enzymatic assays, and analysis of β-amyloid, tau, and synuclein species were used.

**Results:**

The main alterations in DLB and rpDLB, which are more marked in the rapidly progressive forms, include (i) deregulated expression of several mRNAs and proteins of mitochondrial subunits, and reduced activity of complexes I, II, III, and IV of the mitochondrial respiratory chain; (ii) reduced expression of selected molecules involved in energy metabolism and increased expression of enzymes involved in purine metabolism; (iii) abnormal expression of nucleolar proteins, rRNA18S, genes encoding ribosomal proteins, and initiation factors of the transcription at the ribosome; (iv) discrete inflammation; and (v) marked deregulation of brain ORs and TASRs, respectively. Severe mitochondrial dysfunction involving activity of four complexes, minimal inflammatory responses, and dramatic altered expression of ORs and TASRs discriminate DLB from Alzheimer’s disease. Altered solubility and aggregation of α-synuclein, increased β-amyloid bound to membranes, and absence of soluble tau oligomers are common in DLB and rpDLB. Low levels of soluble β-amyloid are found in DLB. However, increased soluble β-amyloid 1–40 and β-amyloid 1–42, and increased TNFα mRNA and protein expression, distinguish rpDLB.

**Conclusion:**

Molecular alterations in frontal cortex in DLB involve key biochemical pathways such as mitochondria and energy metabolism, protein synthesis, purine metabolism, among others and are accompanied by discrete innate inflammatory response.

## Introduction

Dementia with Lewy bodies (DLB) is the second most common neurodegenerative dementia in the elderly, clinically manifested by fluctuating cognition with pronounced variation in attention and alertness, recurrent visual hallucinations which are typically well formed and detailed, and spontaneous motor features of parkinsonism; repeated falls, syncope, transient loss of consciousness, systematized delusions, hallucinations in other modalities, and neuroleptic sensitivity are not uncommon (1–4). These symptoms are preceded by rapid eye movement sleep behavior disorder, psychiatric symptoms, loss of smell, and dysautonomia, together with occipital hypo-metabolism, hallucinations, and cognitive impairment (5, 6).

Dementia with Lewy bodies is pathologically characterized by Lewy bodies and Lewy neurites in the brainstem, limbic system, and cortical areas (2, 7, 8). The main pathological change is the production and accumulation, in Lewy bodies and neurites, of abnormal α-synuclein, which is phosphorylated, nitrated, and truncated, has abnormal solubility, prompts the production of oligomeric species, aggregates into fibrils and is ubiquitinated (9–19). For these reasons, DLB is classified among α-synucleinopathies with Lewy bodies or Lewy body diseases (LBDs), together with Parkinson’s disease (PD) (11). Other changes in DLB are neuron loss, microvacuolation, and Alzheimer’s disease (AD) pathology distinguished by β-amyloid deposition in the form of diffuse and senile plaques, as well as early changes of neurofibrillary tangle (NFT) pathology (8).

Whether the amount of α-synuclein pathology (i.e., Lewy bodies and neurites) in cerebral cortex is predictable of dementia in LBDs is a matter of controversy (20–23). Cholinergic and dopaminergic denervation of the neocortex probably accounts at least in part for cognitive deficits in LBDs (24–29). Concomitant pathologies have also been suggested to explain variations in the degree of cognitive impairment in DLB (30–32).

However, deficits in neurotransmission are not limited to dopaminergic and cholinergic systems. Synapses are primarily damaged in the neocortex in DLB (33–38). Synaptic damage is probably related to toxic α-synuclein oligomers and pore formation (39, 40). Moreover, synaptic alterations are accompanied by abnormalities in neurotransmitter signaling (41, 42) in a way similar to that reported in other α-synucleinopathies (43–45).

Additional molecular alterations converge in the pathogenesis of DLB, including impaired autophagy and ubiquitin-proteasome system of protein (46–50), as well as altered responses to protein misfolding (51). Preliminary studies have shown impaired mitochondrial activity and oxidative damage involving proteins, lipids, and DNA in the neocortex in DLB (52, 53); α-synuclein is one of the targets of oxidative damage in the frontal cortex in DLB (54).

The average survival time for typical DLB from the beginning of symptoms is about 5–8 years (55). However, some cases have a rapid course and are considered clinically to be in the group of rapidly progressive dementias (56, 57). DLB with rapid progression has been named rapid DLB (rpDLB) (55, 58). The mean duration of rpDLB is about 9 months; delirium, visual hallucinations, delusions, and fluctuating cognitive impairment, followed by parkinsonism and myoclonus, are the predominant symptoms (58).

Our hypothesis is that alterations of several metabolic pathways converge in the pathogenesis of DLB and that impaired mitochondria and energy metabolism, purine metabolism, protein synthesis, and inflammation may be important factors in the pathogenesis of DLB. In the same line, although neuropathological studies have not shown differences between DLB and rpDLB (58), biochemical alterations probably discriminate between DLB and rpDLB.

The present DLB-centered study was undertaken to analyze (i) levels of selected mRNAs and proteins of subunits of the five mitochondrial complexes and genes linked to energy metabolism; (ii) expression of genes encoding enzymes involved in purine metabolism; (iii) mRNA and protein expression of selected nucleolar proteins, rRNAs, and genes encoding ribosomal proteins, and protein expression of initiation and elongation factors of protein transcription at the ribosome; (iv) cytokines and mediators of the inflammatory response; and (v) gene expression of particular brain receptors involved in olfaction and taste known to be altered in other neurodegenerative diseases with abnormal protein aggregates. All these pathways were assessed in the frontal cortex of cases with typical course (DLB) and in cases with rapid course (rpDLB) compared with middle-aged (MA) individuals to identify molecular factors linked to Lewy body pathology.

## Materials and Methods

### Human Cases

Brain tissue was obtained from the Institute of Neuropathology HUB-ICO-IDIBELL Biobank and the Hospital Clinic-IDIBAPS Biobank following the guidelines of Spanish legislation on this matter (Real Decreto de Biobancos 1716/2011) and approval of the local ethics committees. Processing of brain tissue has been detailed elsewhere (59, 60). The postmortem interval between death and tissue processing was between 3 and 15.30 h. One hemisphere was immediately cut in coronal sections, 1 cm thick, and selected areas of the encephalon were rapidly dissected, frozen on metal plates over dry ice, placed in individual air-tight plastic bags and stored at −80°C until use for biochemical studies. The other hemisphere was fixed by immersion in 4% buffered formalin for 3 weeks for morphological studies. Neuropathological diagnosis in all cases was based on the routine study of 20 selected de-waxed paraffin sections of representative regions of the cerebral cortex, diencephalon, thalamus, brain stem, and cerebellum, which were stained with hematoxylin and eosin, and Klüver-Barrera, or processed for immunohistochemistry for microglia (antibodies Iba1 and CD68), glial fibrillary acidic protein, β-amyloid with antibodies Aβ clone 6 F/3D (diluted 1:50, Dako, Carpinteria, CA, USA), Aβ40 (diluted 1:100, Merck Millipore, Billerica, MA, USA), and Aβ42 (diluted 1:50, Merck Millipore), phospho-tau (clone AT8), α-synuclein, TDP-43, ubiquitin, and p62 using EnVision + System peroxidase (Dako), and diaminobenzidine and H_2_O_2_. DLB cases (*n* = 13) were neuropathologically categorized following current staging classifications for LBD (22, 61, 62), stages of of NFT pathology (21, 63), and phases of AD-related β-amyloid plaques (64), and a final ABC score was assigned according to current consensus guidelines (65). Neuropathological data were scored by two independent observers. Based on clinical criteria DLB cases were categorized as typical DLB (DLB, *n* = 9: eight men, one woman, age 76.4 ± 5.7 years) or rapid DLB (rpDLB, *n* = 4, two men and two women, age 73.7 ± 2.2) on the basis of the natural clinical course. rpDLB here was defined as 2 years or less of disease duration from the first symptom to death (58). Cases with associated pathologies such as vascular diseases excepting mild atherosclerosis and arteriolosclerosis, TDP-43 proteinopathy, infections of the nervous system, brain neoplasms, systemic and central immune diseases, metabolic syndrome, and hypoxia were excluded from the present study. MA cases (*n* = 12: seven men and five women, age 59.9 ± 15.59) had not suffered from neurologic, psychiatric, or metabolic diseases (including metabolic syndrome) and did not have abnormalities in the neuropathological examination excepting stages I–II of NFT pathology and phases 1–2 of β-amyloid plaques.

Quantification of β-amyloid burden was carried out with a Nikon Eclipse E800 microscope (4× objective; Nikon Imaging Inc., Tokyo, Japan). The cortical total Aβ burden was calculated as the percentage of the area of Aβ deposition in plaques with respect to the total area in 9–10 pictures taken at random from frontal cortex in every case. β-Amyloid quantification was assessed using the Adobe Photoshop CS5 software (Adobe Systems Inc., San Jose, CA, USA).

A summary of cases is shown in Table 1.

**Table 1:** **Summary of cases used in the present study**.

No	Diagnosis	Gender	Age	PM delay	RIN	PCR	WB	ELISA	MA	MI
1	MA	Male	64	8 h 30 min	7.7	X	X	X		
2	MA	Male	56	5 h	7.1	X	X	X	X	X
3	MA	Male	67	5 h	7	X	X	X	X	
4	MA	Male	62	3 h	7.2	X	X	X	X	X
5	MA	Male	52	4 h 40 min	7.9	X	X	X		X
6	MA	Male	30	4 h 10 min	8.4	X	X	X	X	
7	MA	Male	53	3 h	7.7	X	X	X		X
8	MA	Female	49	7 h	8.2	X	X	X		
9	MA	Female	75	3 h	6.5	X	X	X		
10	MA	Female	46	9 h 35 min	7.2	X	X	X	X	
11	MA	Female	86	4 h 15 min	8.4	X	X	X		
12	MA	Female	79	3 h 35 min	8	X	X	X		
13	DLB	Male	81	7 h	5.3	X	X	X		
14	DLB	Female	78	5 h	5.7	X	X	X		X
15	DLB	Male	76	5 h 10 min	5.2	X	X	X		X
16	DLB	Male	83	9 h	5.2	X	X	X	X	X
17	DLB	Male	78	8 h 30 min	6.3	X	X	X		
18	DLB	Male	64	8 h 15 min	7	X	X	X	X	
19	DLB	Male	80	8 h	6.1	X	X	X		X
20	DLB	Male	77	7 h 20 min	6.4	X	X	X		
21	DLB	Male	71	9 h	7	X	X	X	X	
22	rpDLB	Female	75	13 h 30 min	*5*.6	X	X	X	X	X
23	rpDLB	Male	76	6 h 30 min	5.5	X	X	X	X	X
24	rpDLB	Male	71	5 h	5	X	X	X	X	X
25	rpDLB	Female	73	15 h 30 min	6.1	X	X	X		X

*MA, middle-aged cases with no neurological disease and neuropathological lesions limited to Alzheimer’s disease-related pathology stages I–II/0–A of Braak and phases 1–2 of β-amyloid plaques; mean age ± SD was 59.92 ± 15.60. DLB cases were older with no differences between DLB (76.44 ± 5.77) and rpDLB (73.75 ± 2.22). DLB, the term DLB is used to name clinically and neuropathologically verified dementia with Lewy bodies, whereas rpDLB indicates DLB with rapid course. PM delay, postmortem delay; RIN, RNA integrity number; PCR, implies the use of these samples for gene expression studies; WB, cases analyzed with western blotting; ELISA, enzyme-linked immunosorbent assay; MA, mitochondrial enzyme activities; MI, study of α-synuclein oligomeric species in total homogenate fractions. RIN median: 7. Ratio RIN min/max: 5/8.4. PM delay median: 6 h 30 m. PM delay: min/max: 3/15 h 30 m*.

### RNA Purification

Purification of RNA from the right frontal cortex area 8 was carried out using RNeasy Lipid Tissue Mini Kit (Qiagen, Hilden, Germany) following the protocol provided by the manufacturer combined with DNase digestion to avoid extraction and later amplification of genomic DNA. The concentration of each sample was obtained from A260 measurements with NanoDrop 2000 spectrophotometer (Thermo Scientific, Waltham, MA, USA). RNA integrity was tested using the Agilent 2100 BioAnalyzer (Agilent, Santa Clara, CA, USA) (66). Values of RNA integrity number (RIN) are shown in Table 1. Special care was taken to assess pre-mortem and postmortem factors, which may interfere with RNA processing (67).

Bivariate analyses were carried out to detect association of our variables with potential confounding factors (age, postmortem delay, and RIN) using Spearman or Pearson correlations for quantitative variables. Stastical analysis was performed with GraphPad Prism version 5.00 and SPSS 19. Postmortem delay had no effect on RIN values in the present series.

### Retrotranscription Reaction

Retrotranscription reaction of RNA samples was carried out with the High-Capacity cDNA Archive kit (Applied Biosystems, Foster City, CA, USA) following the guidelines provided by the supplier, and using Gene Amp^®^ 9700 PCR System thermocycler (Applied Biosystems). A parallel reaction for one RNA sample was processed in the absence of reverse transcriptase to rule out DNA contamination.

### Real-time PCR

Real-time quantitative PCR (RT-qPCR) assays were conducted in duplicate on 1,000 ηg of cDNA samples obtained from the retrotranscription reaction, diluted 1:20 in 384-well optical plates (Kisker Biotech, Steinfurt, GE) utilizing the ABI Prism 7900 HT Sequence Detection System (Applied Biosystems). Parallel amplification reactions were carried out using 20× TaqMan Gene Expression Assays and 2× TaqMan Universal PCR Master Mix (Applied Biosystems) (66). Genes analyzed with the corresponding abbreviations and TaqMan probes used in the study are shown in Table 2.

**Table 2:** **Abbreviations, full names, and TaqMan probes used to assess gene expression in the frontal cortex of MA and DLB cases in the present study**.

Gene	Full name	Reference
**Housekeeping genes**		
*GUS-B*	β-glucuronidase	Hs00939627_m1
*XPNPEP1*	X-prolylaminopeptidase (aminopeptidase P) 1	Hs00958026_m1
*AARS*	Alanyl-tRNA synthetase	Hs00609836_m1
*HPRT*	Hypoxanthine phosphoribosyltransferase 1	Hs_02800695_m1
**Genes encoding proteins of mitochondria and energy metabolism-related molecules**		
*NDUFA2*	NADH dehydrogenase (ubiquinone) 1 alpha subcomplex, 2, 8 kDa	Hs00159575_m1
*NDUFA7*	NADH dehydrogenase (ubiquinone) 1 alpha subcomplex, 7, 14.5kDa	Hs01561430_m1
*NDUFA10*	NADH dehydrogenase (ubiquinone) 1 alpha subcomplex, 10, 42kDa	Hs01071117_m1
*NDUFB3*	NADH dehydrogenase (ubiquinone) 1 beta subcomplex, 3, 12 kDa	Hs00427185_m1
*NDUFB7*	NADH dehydrogenase (ubiquinone) 1 beta subcomplex, 7, 18 kDa	Hs00188142_m1
*NDUFB10*	NADH dehydrogenase (ubiquinone) 1 beta subcomplex, 10, 22 kDa	Hs00605903_m1
*NDUFS7*	NADH dehydrogenase (ubiquinone) Fe-S protein 7, 20 kDa	Hs00257018_m1
*NDUFS8*	NADH dehydrogenase (ubiquinone) Fe-S protein 8, 23 kDa	Hs00159597_m1
*SDHB*	Succinate dehydrogenase complex, subunit B, iron sulfur (Ip)	Hs00268117_m1
*UQCRB*	Ubiquinol-cytochrome c reductase binding protein	Hs00559884_m1
*UQCR11*	Ubiquinol-cytochrome c reductase, complex III subunit XI	Hs00907747_m1
*COX7A2L*	Cytochrome c oxidase subunit VIIa polypeptide 2 like	Hs00190880_m1
*COX7C*	Cytochrome c oxidase subunit VIIc	Hs01595220_g1
*ATP5D*	ATP synthase, H+ transporting, mitochondrial F1 complex, delta subunit	Hs00961521_m1
*ATP5G2*	ATP synthase, H+ transporting, mitochondrial Fo complex, subunit C2	Hs01096582_m1
*ATP5H*	ATP synthase, H+ transporting, mitochondrial Fo complex, subunit d	Hs01046892_gH
*ATP5L*	ATP synthase, H+ transporting, mitochondrial Fo complex, subunit G	Hs00538946_g1
*ATP5O*	ATP synthase, H+ transporting, mitochondrial F1 complex, O subunit	Hs00426889_m1
*ATP2B3*	ATPase, Ca++ transporting, plasma membrane 3	Hs00222625_m1
*ATP2B4*	ATPase, Ca++ transporting, plasma membrane 4	Hs00608066_m1
*ATP4A*	ATPase, H+/K+ exchanging, alpha polypeptide	Hs00167575_m1
*ATP6V0A1*	ATPase, H+ transporting, lysosomal V0 subunit a1	Hs00193110_m1
*ATP6V0B*	ATPase, H+ transporting, lysosomal 21kDa, V0 subunit b	Hs01072388_m1
*ATP6V1H*	ATPase, H+ transporting, lysosomal 50/57kDa, V1 subunit H	Hs00977530_m1
*FAM82A2*	Family with sequence similarity 82, member A2	Hs00216746_m1
*LHPP*	Phospholysine phosphohistidine inorganic pyrophosphate phosphatase	Hs00383379_m1
*SLC6A6*	Solute carrier family 6 (neurotransmitter transporter, taurine), member 6	Hs00161778_m1
*SLC25A31*	Solute carrier family 25 (mitochondrial carrier; adenine nucleotide translocator), member 31	Hs00229864_m1
*TOMM40*	Translocase of outer mitochondrial membrane 40 homolog (yeast)	Hs01587378_mH
*ZNF642*	Zinc finger protein 642	Hs01372953_m1
**Purine metabolism genes**		
*ADA*	Adenosine deaminase	Hs01110945_m1
*AK1*	Adenylate kinase (AK) 1	Hs00176119_m1
*AK2*	AK 2	Hs01123132_g1
*AK4*	AK 4	Hs03405743_g1
*AK5*	AK 5	Hs00952786_m1
*AK7*	AK 7	Hs00330574_m1
*APRT*	Adenine phosphoribosyltransferase	Hs00975725_m1
*DGUOK*	Deoxyguanosine kinase	Hs00176514_m1
*ENTPD1*	Ectonucleoside triphosphate diphosphohydrolase 1	Hs00969559_m1
*ENTPD2*	Ectonucleoside triphosphate diphosphohydrolase 2	Hs00154301_m1
*ENTPD3*	Ectonucleoside triphosphate diphosphohydrolase 3	Hs00928977_m1
*NME1*	Non-metastatic cells 1, protein expressed in (nucleoside-diphosphate kinase)	Hs02621161_s1
*NME3*	Non-metastatic cells 3, protein expressed in (nucleoside-diphosphate kinase)	Hs01573874_g1
*NME4*	Non-metastatic cells 4, protein expressed in (nucleoside-diphosphate kinase)	Hs00359037_m1
*NME5*	Non-metastatic cells 5, protein expressed in (nucleoside-diphosphate kinase)	Hs00177499_m1
*NME6*	Non-metastatic cells 6, protein expressed in (nucleoside-diphosphate kinase)	Hs00195083_m1
*NME7*	Non-metastatic cells 7, protein expressed in (nucleoside-diphosphate kinase)	Hs00273690_m1
*NT5C*	5′, 3′-nucleotidase, cytosolic	Hs00274359_m1
*NT5E*	5′-nucleotidase, ecto (CD73)	Hs00159686_m1
*PNP*	Purine nucleoside phosphorylase	Hs01002926_m1
*POLR3B*	Polymerase (RNA) III (DNA directed) polypeptide B	Hs00932002_m1
*PRUNE*	Prune homolog (*Drosophila*)	Hs00535700_m1
**Nucleolar, rRNAs, and genes encoding ribosomal protein**		
*NCL*	Nucleolin	Hs01066668_m1
*NPM1*	Nucleophosmin (nucleolar phospho-protein B23, numatrin)	Hs02339479_m1
*NPM3*	Nucleophosmin/nucleoplasmin 3	Hs00199625_m1
*rRNA 28S*	RNA, 28S ribosomal 5	Hs03654441_s1
*rRNA 18S*	Eukaryotic 18S rRNA	Hs99999901_s1
*UBTF*	Upstream binding transcription factor, RNA polymerase I	Hs01115792_g1
*RPL5*	Ribosomal protein L5	Hs_03044958_g1
*RPL7*	Ribosomal protein L7	Hs_02596927_g1
*RPL21*	Ribosomal protein L21	Hs_00823333_s1
*RPL22*	Ribosomal protein L22	Hs_01865331_s1
*RPL23A*	Ribosomal protein L23A	Hs_01921329_g1
*RPL26*	Ribosomal protein L26	Hs_00864008_m1
*RPL27*	Ribosomal protein L27	Hs_03044961_g1
*RPL30*	Ribosomal protein L30	Hs_00265497_m1
*RPL31*	Ribosomal protein L31	Hs_0101549_g1
*RPS3A*	Ribosomal protein S3A	Hs_00832893_sH
*RPS5*	Ribosomal protein S5	Hs_00734849_g1
*RPS6*	Ribosomal protein S6	Hs_04195024_g1
*RPS10*	Ribosomal protein S10	Hs_01652370_gH
*RPS13*	Ribosomal protein S13	Hs_01011487_g1
*RPS16*	Ribosomal protein S16	Hs_01598516_g1
*RPS17*	Ribosomal protein S17	Hs_00734303_g1
**Cytokines and mediators of the inflammatory responses**		
*C1QL1*	Complement component 1, q subcomponent 1	Hs00198578_m1
*C1QTNF7*	C1q and tumor necrosis factorY related protein 7	Hs00230467_m1
*C3AR1*	Complement component 3a receptor 1	Hs00377780_m1
*CLEC7A*	C-type lectin domain family 7, member A	Hs01124746_m1
*CSF1R*	Colony-stimulating factor 1 receptor	Hs00911250_m1
*CSF3R*	Colony-stimulating factor 1 receptor	Hs00167918_m1
*CST7*	Cystatin F (leukocystatin)	Hs00175361_m1
*CTSC*	Cathepsin C	Hs00175188_m1
*CTSS*	Cathepsin S	Hs00356423_m1
*IL1B*	Interleukin-1B	Hs01555410_m1
*IL6*	Interleukin-6	Hs00985639_m1
*IL6ST*	Interleukin-6 signal transducer	Hs00174360_m1
*IL8*	Interleukin-8	Hs00174103_m1
*IL10*	Interleukin-10	Hs00961622_m1
*IL10RA*	Interleukin-10 receptor A	Hs00155485_m1
*IL10RB*	Interleukin-10 receptor B	Hs00609836_m1
*TGFA1*	Transforming growth factor-A1	Hs00998133_m1
*TGFA2*	Transforming growth factor-A2	Hs00234244_m1
*TLR4*	Toll-like receptor 4	Hs01060206_m1
*TLR7*	Toll-like receptor 7	Hs00152971_m1
*TNF*	Tumor necrosis factor	Hs01113624_m1
*TNFRSF1A*	Tumor necrosis factor receptor superfamily member 1a	Hs01042313_m1
**Olfactory receptor (OR) and taste receptor (TASR) genes**		
*OR2D2*	OR, family 2, subfamily D, member 2	Hs00999189_s1
*OR2J3*	OR, family 2, subfamily J, member 3	Hs01943871_g1
*OR2L13*	OR, family 2, subfamily L, member 13	Hs00380097_m1
*OR2T1*	OR, family 2, subfamily T, member 1	Hs01661970_s1
*OR2T33*	OR, family 2, subfamily T, member 33	Hs04230793_gH
*OR4F4*	OR, family 4, subfamily F, member 4	Hs03406040_gH
*OR6F1*	OR, family 6, subfamily F, member 1	Hs01054972_s1
*OR10G8*	OR, family 10, subfamily G, member 8	Hs01943074_g1
*OR11H1*	OR, family 11, subfamily H, member 1	Hs03406084_gH
*OR51E1*	OR, family 51, subfamily E, member 1	Hs02339849_s1
*OR52H1*	OR, family 52, subfamily H, member 1	Hs01661724_s1
*OR52L1*	OR, family 52, subfamily L, member 1	Hs02339119_g1
*OR52M1*	OR, family 52, subfamily M, member 1	Hs01098608_s1
*TAS2R4*	TASR, type 2, member 4	Hs00249946_s1
*TAS2R5*	TASR, type 2, member 5	Hs01549633_s1
*TAS2R10*	TASR, type 2, member 10	Hs00256794_s1
*TAS2R13*	TASR, type 2, member 13	Hs01059805_s1
*TAS2R14*	TASR, type 2, member 14	Hs00256800_s1
*TAS2R50*	TASR, type 2, member 50	Hs00604351_s1

The selection of probes was based on criteria covering a larger project geared to analyzing similar expression of the same molecules related to inflammation, mitochondria, energy metabolism, purine metabolism, protein synthesis and selected receptors in several diseases including AD, PD, Creutzfeldt-Jakob’s disease, certain tauopathies, in addition to DLB, in all cases using the same probes and methods in an attempt to learn about commonalities and differences among these diseases in a particular brain region. Our idea is that all these pathways are altered in most neurodegenerative diseases with abnormal protein aggregates, but alterations are region-, stage-, and disease-specific.

Parallel assays for each sample were carried out using β-glucuronidase (*GUS-*β), X-prolyl aminopeptidase (aminopeptidase P) 1 (*XPNPEP1*), *AARS* (alanyl-transfer RNA synthase), and *HPRT* (hypoxanthine-guanine phosphoribosyltransferase) probes for normalization. The selection of these housekeeping genes was based on previous data showing low vulnerability in the brain of several human neurodegenerative diseases (68, 69). The reactions were performed using the following parameters: 50°C for 2 min, 95°C for 10 min, 40 cycles at 95°C for 15 s, and 60°C for 1 min. TaqMan PCR data were captured using the Sequence Detection Software (SDS version 2.2, Applied Biosystems). Subsequently, threshold cycle (CT) data for each sample were analyzed with the double-delta CT (ΔΔCT) method (66). First, delta CT (ΔCT) values were calculated as the normalized CT values for each target gene in relation to the mean values of *GUS-*β, *XPNPEP1*, AARS, and *HPRT*. Second, ΔΔCT values were obtained with the ΔCT of each sample minus the mean ΔCT of the population of control samples (calibrator samples). The fold change was determined using the equation 2^−ΔΔCT^.

### Gel Electrophoresis and Western Blotting from Total Homogenate

Tissue was processed as reported elsewhere (66). A total of 0.1 g of tissue of samples from frontal cortex area 8 were lysed with a glass homogenizer in Mila lysis buffer (0.5M Tris at pH 7.4 containing 0.5 methylenediaminetetraacetic acid at pH 8.0, 5M NaCl, 0.5% Na deoxycholic acid, 0.5% Non-idet P-40, 1 mM phenylmethylsulfonyl fluoride, bi-distilled water, and protease and phosphatase inhibitor cocktails (Roche Molecular Systems, Pleasanton, CA, USA)), and then centrifuged for 15 min at 13,000 rpm at 4°C (Ultracentrifuge Beckman with 70Ti rotor, CA, USA). Protein concentration was measured with SmartspectTMplus spectrophotometer (Bio-Rad, CA, USA) using the Bradford method (Merck, Darmstadt, Germany). Samples containing 15 μg of protein and the standard Precision Plus ProteinTM Dual Color (Bio-Rad) were loaded onto 10–15% acrylamide gels. Proteins were separated with sodium dodecylsulfate-polyacrylamide gel electrophoresis (SDS-PAGE) and electrophoretically transferred to nitrocellulose membranes using the Trans-Blot^®^TurboTM transfer system (Bio-Rad) at 200 mA/membrane for 40 min. Non-specific bindings were blocked by incubation with 5% milk in Tris-buffered saline (TBS) containing 0.1% Tween for 1 h at room temperature. After washing, the membranes were incubated at 4°C overnight with one of the primary antibodies (Table S1 in Supplementary Material) in TBS containing 3% albumin and 0.1% Tween. These membranes were incubated for 1 h with the appropriate HRP-conjugated secondary antibody (1:2,000, Dako, Glostrup, Denmark), and the immune complexes were revealed with a chemiluminescence reagent (ECL, Amersham, GE Healthcare, Buckinghamshire, UK). Monoclonal anti-β-actin antibody diluted 1:30,000 (β-Actin, A5316; Sigma-Aldrich, St. Louis, MO, USA) was blotted to control protein loading.

### Mitochondrial Enzymatic Activities

The activities of mitochondrial complexes I, II, III, IV, and V were analyzed using commercial kits following the manufacturers’ instructions (mitochondrial complex I, II, IV, and V: Novagen, Merck Biosciences, Darmstadt, Germany; mitochondrial complex III: MyBiosource, CA, USA). Isolation of mitochondria was carried out as reported in detail elsewhere (70). Briefly, mitochondria were extracted from frozen human brain tissue (100 mg) under ice-cold conditions. Tissues were minced in ice-cold isolation buffer (IB), and then homogenized and centrifuged at 1,000 × *g* for 10 min. The supernatant (S1) was conserved. The pellet was washed with two volumes of IB and centrifuged again under the same conditions. This last supernatant (S2) was combined with S1. Centrifugation at 10,000 × *g* for 10 min at 4°C resulted in the mitochondria-enriched pellet. The pellet was finally re-suspended in one volume of IB and stored at −80°C. Protein concentration was measured by Smartspect™ plus spectrophotometer (Bio-Rad, CA, USA) using the Bradford method (Merck, Darmstadt, Germany). Twenty-five micrograms of mitochondria was loaded into each well. Activity of citrate synthase was evaluated following validated protocols (71) with slight modifications. The activity of citrate synthase was determined as the rate of reduction of DTNB [5′, 5′-dithiobis (2-nitrobenzoic acid)] to thionitrobenzoic acid at 412 nm. For this purpose, 25 μg of mitochondria was added to a 1ml mixture containing 500 μl of Tris (200mM, pH 8.0) with Triton X-100 [0.2% (vol/vol)], 100 μl of DTNB, and 30 μl of 10mM Acetyl CoA, and then the final volume was adjusted to 950 l with distilled water. The reaction was started by the addition of 50 l of 10 mM oxalacetic acid. The increase in absorbance at 412 nm was read for 3 min at room temperature with a DU 800UV/Visible spectrophotometer (Beckman Coulter, CA, USA) in 1 ml polystyrene or methacrylate cuvettes (72).

### Concentration of β-Amyloid 1–40 (Aβ40) and β-Amyloid 1–42 (Aβ42)

Frozen brain samples were homogenized in TBS buffer composed of 140 mM NaCl, 3 mM KCl, 25 mM Tris–HCl pH 7.4, and 5 mM ethylene-diamine-tetra-acetic acid (EDTA) with a cocktail of protease inhibitors (Roche Molecular Systems, Pleasanton, CA, USA), and ultra-centrifuged at 100,000 × *g* for 1 h at 4°C. The supernatant was the soluble fraction used for amyloid quantification, and the protein of this fraction was measured with BCA. The detection and measurement of β-amyloid 1–40 (Aβ40) and β-amyloid 1–42 (Aβ42) were carried out by enzyme-linked immune-absorbent assay with the corresponding detection kits (Invitrogen, Camarillo, CA, USA), following the instructions of the supplier. TBS-soluble Aβ40 and Aβ42 levels were normalized to the total amount of protein from each individual sample (73).

### Quantification of Membrane-Associated β-Amyloid

Frozen samples were homogenized in TBS with a cocktail of protease and phosphatase inhibitors (Roche Molecular Systems). Homogenates were centrifuged at 14,000 × *g* for 30 min at 4°C. The pellet was re-suspended in 2% SDS and centrifuged at 14,000 × *g* for 30 min at 4°C. The supernatant was membrane-associated Aβ and the protein of this fraction was measured with BCA method (Thermo Scientific, USA). Proteins were separated in SDS-polyacrylamide gel electrophoresis. Thirty-five micrograms of protein was loaded onto a precast NuPAGE 4–12% Bis-Tris gel system (Invitrogen, MA, USA) with MES buffer (Invitrogen, MA, USA). The proteins were transferred to nitrocellulose membranes, 200 mA/membrane, for 90 min. Then membranes were boiled with PBS 1× for 15 min, and non-specific bindings were blocked by incubation in 5% non-fat dry milk in TBS containing 0.2% Tween for 1 h at room temperature. After washing, the membranes were incubated at 4°C overnight with primary antibodies to human amyloid-beta protein, clone: 4G8 (1:500, Signet, MA, USA) and 6E10 (1:2,000, Covance, NJ, USA) in TBS containing 5% albumin and 0.2% Tween. After washing, the membranes were incubated at 4°C overnight with the primary antibody to human amyloid-beta protein, clone: 4G8 (1:500, Signet, MA, USA) in TBS containing 5% albumin and 0.2% Tween. Membranes were then incubated for 1 h with the appropriate HRP-conjugated secondary antibody (1:2,000, Dako, DK), and the immune complexes were visualized with a chemiluminescence reagent (ECL, Amersham, GB). β-Actin was used as a control of protein loading (73). Using this protocol, membrane-enriched fractions may also contain small amounts of TBS-soluble β-amyloid.

### Oligomeric Tau Species

Frozen samples were homogenized in lysis buffer: 100 mM Tris (pH 7.0), 100 mM NaCl, 10 mM EDTA, 0.5% NP-40, and 0.5% sodium deoxycholate plus protease and phosphatase inhibitors (Roche Molecular Systems). After centrifugation at 14,000 × *g* for 20 min at 4°C (Ultracentrifuge Beckman with 70Ti rotor), supernatants were quantified for protein concentration (BCA), mixed with SDS-PAGE sample buffer, boiled, and separated to 8% SDS-PAGE gels. The proteins were transferred to nitrocellulose membranes, 200 mA/membrane, for 90 min. The membranes were blocked with 5% non-fat milk in TBS containing 0.2% Tween for 1 h at room temperature. After washing, the membranes were incubated at 4°C overnight with the primary antibody anti-tau-5 (1:1,000, Thermo-Fisher, MA, USA) in TBS containing 5% albumin and 0.2% Tween. Membranes were then incubated for 1 h with the HRP-conjugated secondary anti-mouse antibody (1:2,000, Dako, DK), and the immune complexes were visualized with ECL. β-Actin was used as a control of protein loading (74). No attempt was made to analyze sarkosyl-insoluble fractions as oligomeric tau species are visualized with the utilized protocol and the identification of tau bands was not an objective of the present study.

### α-Synuclein Oligomeric Species in Total Homogenate Fractions

Frozen samples were homogenized in a glass homogenizer, in 750 μl of ice-cold PBS+ (sodium phosphate buffer pH 7.0, plus protease inhibitors), sonicated, and centrifuged at 2,700 × *g* at 4°C for 10 min. The pellet was discarded and the resulting supernatant was ultra-centrifuged at 43,000 × *g* at 4°C for 1 h. The supernatant (S2) was kept as the PBS-soluble fraction. The resulting pellet was re-suspended in a solution of PBS, pH 7.0, containing 0.5% sodium deoxycholate, 1% Triton and 0.1% SDS, and this was ultra-centrifuged at 43,000 × *g* at 4°C for 1 h. The resulting supernatant (S3) was kept as the deoxycholate-soluble fraction. The corresponding pellet was re-suspended in a solution of 2% SDS in PBS and maintained at room temperature for 30 min. Afterward, the samples were centrifuged at 133,000 × *g* at 25°C for 1 h and the resulting supernatant (S4) was the SDS-soluble fraction. Equal amounts of each fraction were mixed with reducing sample buffer and processed in parallel for 10% SDS-PAGE electrophoresis and western blotting. Membranes were incubated with anti-α-synuclein oligomer-specific antibody (Agrisera, Vännäs, Sweden) at a dilution of 1:1,000. The protein bands were visualized with the ECL method (66).

### Statistical Analysis

The normality of distribution of the mean fold-change values obtained by RT-qPCR for every region and stage between controls and DLB cases was analyzed with the Kolmogorov–Smirnov test. The non-parametric Kruskal–Wallis test was performed to compare groups when the samples did not follow a normal distribution, whereas the one-way ANOVA with *post hoc* Tukey’s range test for multiple comparisons was used for normal variables. Statistical analysis was performed with GraphPad Prism version 5.01 (La Jolla, CA, USA) and Statgraphics Statistical Analysis and Data Visualization Software version 5.1 (Warrenton, VA, USA). Differences between groups were considered statistically significant at *p*-values: *p < 0.05, **p < 0.01, and ***p < 0.001.

Because of the large number of parameters and regions analyzed, multiple comparison tests and false discovery rate might be employed (75, 76). However, due to the limited number of cases, the possibility to obtain significant results minimizes (77). Therefore, present quantitative results must be considered exploratory.

Densitometry of western blot bands was assessed with the TotalLab program (TotalLab Quant, Newcastle, UK) and then subsequently analyzed with GraphPad Prism, Statgraphics Statistical Analysis and Data Visualization Software version 5.1 (VA, USA) by one-way ANOVA with *post hoc* Tukey’s range test for multiple comparisons. Differences were considered statistically significant at *p*-values: *p < 0.05, **p < 0.01, and ***p < 0.001.

The enzymatic activities for each mitochondrial complex were expressed as a rate of nanomoles per minute per milligram of mitochondrial protein per protein concentration normalized with the mitochondrial complex activity rate of citrate synthase activity. Data were presented as mean ± SEM for all the experiments. All the data were analyzed with Student’s *t*-test using GraphPad Prism version 5.01 (La Jolla, CA, USA) and Statgraphics Statistical Analysis and Data Visualization Software version 5.1 (Warrenton, VA, USA). In all experimental procedures the significance level was set at *p < 0.05, **p < 0.01, and ***p < 0.001.

## Results

### General Neuropathological Findings

Brain weight and neuropathological characteristics in the frontal cortex including neuron loss, astrocytic gliosis, microgliosis, spongiosis, diffuse plaques, senile plaques, β-amyloid angiopathy, and α-synuclein aggregates (Lewy bodies and Lewy neurites) were assessed in DLB and rpDLB cases. Thal phase of β-amyloid deposition, Braak stages of NFT pathology, CERAD global, ABC classification, and LBD stage were also considered to frame AD- and LBD-related pathology in every case. Results are summarized in Table 3. A remarkable observation was the discrete microglial response, as revealed with Iba1 and CD68 antibodies, in the frontal cortex in DLB and rpDLB.

**Table 3:** **Summary of the main neuropathological findings of MA (cases 1–12), DLB (cases 13–21), and rpDLB cases (cases 22–25) in the present series**.

Case	Brain weight	rDLB	*N* loss	Astrocytosis	Microglia	Spongiosis	Diffuse plaques	Senile plaques	P-tau	Lewy bodies	Thal phase	Neurofibrillary tangle Braak stage	CERAD	ABC	Lewy body diseases stage
1	NA	–	Absent	Absent	Absent	Absent	Absent	Absent	Isolated	Absent	0	I	0	A0B1C0	0
2	NA	–	Absent	Absent	Absent	Absent	Absent	Absent	Absent	Absent	0	0	0	A0B0C0	0
3	NA	–	Absent	Absent	Absent	Absent	Absent	Absent	Isolated	Absent	0	II	0	A0B1C0	0
4	NA	–	Absent	Absent	Absent	Absent	Absent	Absent	Isolated	Absent	0	II	0	A0B1C0	0
5	NA	–	Absent	Absent	Absent	Absent	Absent	Absent	Absent	Absent	0	0	0	A0B0C0	0
6	NA	–	Absent	Absent	Absent	Absent	Absent	Absent	Absent	Absent	0	0	0	A0B0C0	0
7	NA	–	Absent	Absent	Absent	Absent	Absent	Absent	Absent	Absent	0	0	0	A0B0C0	0
8	NA	–	Absent	Absent	Absent	Absent	Absent	Absent	Absent	Absent	0	0	0	A0B0C0	0
9	NA	–	Absent	Absent	Absent	Absent	Rare	Rare	Isolated	Absent	1	III	0	A1B1C0	0
10	NA	–	Absent	Absent	Absent	Absent	Absent	Absent	Absent	Absent	0	0	0	A0B0C0	0
11	NA	–	Absent	Absent	Absent	Absent	Moderate	Moderate	Sparce	Absent	2	IV	0	A2B2C0	0
12	NA	–	Absent	Absent	Absent	Absent	Moderate	Moderate	Sparce	Absent	2	IV	0	A2B2C0	0
13	1,295	–	Moderate	Moderate	Moderate	Mild	Frequent	Moderate	Isolated	Frequent	4	III	Moderate	A3B2C2	Neocortical
14	795	–	Severe	Severe	Severe	Mild	Frequent	Frequent	Frequent	Moderate	5	VI	Frequent	A3B3C3	Neocortical
15	1,230	–	Mild	Mild	Mild	Mild	Frequent	Moderate	Sparse	Moderate	5	V incipient	Frequent	A3B3C3	Neocortical
16	1,300	–	Mild	Moderate	Moderate	Mild	Frequent	Moderate	Moderate	Frequent	5	IV	Moderate	A3B2C2	Neocortical
17	1,220	–	Mild	Mild	Mild	Mild	Severe	Moderate	Sparse	Frequent	4	IV	Moderate	A3B2C2	Neocortical
18	1,370	–	Mild	Mild	Mild	Mild	Absent	Absent	Absent	Sparse	0	I	0	A0B1C0	Limbic
19	1,280	–	Mild	Moderate	Mild	Mild	Moderate	Moderate	Isolated	Moderate	3	II	Moderate	A2B1C2	Neocortical
20	1,365	–	Mild	Mild	Mild	Mild	Absent	Absent	Absent	Sparse	0	III	0	A0B2C0	Limbic
21	1,300	–	Moderate	Moderate	Moderate	Mild	Moderate	Frequent	Isolated	Frequent	4	II	Moderate	A3B1C2	Neocortical
22	1,120	Yes	Moderate	Moderate	Moderate	Mild	Moderate	Moderate	Sparse	Sparse	4	II	Moderate	A3B1C2	Limbic
23	1,300	Yes	Mild	Mild	Mild	Mild	Moderate	Moderate	Moderate	Sparse	5	V	Moderate	A3B3C2	Limbic
24	1,300	Yes	Mild	Moderate	Moderate	Mild	Absent	Absent	Absent	Sparse	0	0	0	–	Limbic
25	1,240	Yes	Mild	Mild	Mild	Mild	Moderate	Frequent	Sparse	Frequent	5	V incipient	Frequent	A3B3C3	Neocortical

*NA, not available*.

No differences in the amount of fibrillar β-amyloid, considering diffuse and senile plaques, were observed between the two groups; the total plaque burden varied from absent to frequent in both groups. The average percentage of Aβ40 of the total β-amyloid in plaques was 7.24% in DLB and 6.03% in rpDLB, and the percentage of Aβ42 was 68.49% in DLB and 67.70% in rpDLB. NFT pathology varied from stage I to stage VI in DLB and from stage 0 to stage V in rpDLB. NFTs in frontal cortex occurred in two DLB and two rpDLB cases.

Astrocytic gliosis was variable from one case to another and varied from mild to moderate in DLB and rpDLB excepting one DLB in which astrocytic gliosis was considered severe.

A remarkable observation was the discrete microglial response, as revealed with Iba1 and CD68 antibodies, in the frontal cortex in DLB and rpDLB.

Neuropathological findings are summarized in Table 3.

### mRNA Expression Levels of Selected Mitochondrial Subunits and Energy Metabolism-Related Molecules in MA, DLB, and rpDLB

The expression levels of five of twenty-seven genes analyzed was deregulated in DLB when compared with MA cases including increase expression of *ATP5G2* and *ATP5H* involved in mitochondrial complex (*p* < 0.05). Deregulation of energy metabolism genes included decrease in *ATP6VOB* and increase in *ATP4A* and *SLC6A6* (*p* < 0.05). Regarding rpDLB, the expression levels of *ATP6VOB* and *TOMM40*, involved in energy metabolism, was decreased in rpDLB (*p* < 0.01 and *p* < 0.05, respectively) (Table 4). Since the trend of altered gene expression was in the same direction in DLB and rpDLB, no significant differences were observed when comparing DLB and rpDLB (Table 4).

**Table 4:** **mRNA expression of selected subunits of the mitochondrial respiratory chain and genes encoding proteins linked to energy metabolism in MA (*n* = 12), DLB (*n* = 9), and rpDLB (*n* = 4)**.

	Probes	MA	DLB	rpDLB	MA vs DLB	MA vs rpDLB	DLB vs rpDLB
**Mitochondria**							
Complex I	*NDUFA2*	1.02 ± 0.22	1.01 ± 0.09	0.94 ± 0.08	–	–	–
	*NDUFA7*	1.27 ± 0.58	0.89 ± 0.25	1.00 ± 0.11	–	–	–
	*NDUFA10*	1.03 ± 0.27	1.11 ± 0.26	1.19 ± 0.31	–	–	–
	*NDUFB3*	1.02 ± 0.23	0.95 ± 0.24	0.99 ± 0.20	–	–	–
	*NDUFB7*	1.02 ± 0.23	1.19 ± 0.53	1.25 ± 0.13	–	–	–
	*NDUFB10*	1.05 ± 0.35	0.87 ± 0.30	1.19 ± 0.20	–	–	–
	*NDUFS7*	1.09 ± 0.47	0.98 ± 0.26	0.71 ± 0.14	–	–	–
	*NDUFS8*	1.03 ± 0.25	1.19 ± 0.66	1.64 ± 0.25	–	–	–
Complex II	*SDHB*	1.05 ± 0.38	0.84 ± 0.16	0.84 ± 0.16	–	–	–
Complex III	*UQCRB*	1.06 ± 0.40	1.09 ± 0.44	1.00 ± 0.24	–	–	–
	*UQCR11*	1.02 ± 0.19	1.10 ± 0.26	1.28 ± 0.27	–	–	–
Complex IV	*COX7A2L*	1.08 ± 0.41	0.93 ± 0.39	1.07 ± 0.38	–	–	–
	*COX7C*	1.03 ± 0.24	0.89 ± 0.38	0.85 ± 0.24	–	–	–
Complex V	*ATP5D*	1.03 ± 0.24	0.98 ± 0.18	0.93 ± 0.13	–	–	–
	*ATP5G2*	1.07 ± 0.42	0.61 ± 0.23	0.69 ± 0.02	*↓ (0.0118)	–	–
	*ATP5H*	1.02 ± 0.21	0.80 ± 0.18	0.82 ± 0.05	*↓ (0.0281)	–	–
	*ATP5L*	1.03 ± 0.26	1.00 ± 0.23	1.01 ± 0.06	–	–	–
	*ATP5O*	1.02 ± 0.18	0.86 ± 0.22	0.95 ± 0.14	–	–	–
**Energy metabolism-related molecules**							
	*ATP2B3*	1.03 ± 0.25	0.99 ± 0.45	1.03 ± 0.33	–	–	–
	*ATP2B4*	1.01 ± 0.18	0.97 ± 0.27	0.83 ± 0.30	–	–	–
	*ATP4A*	1.11 ± 0.51	2.15 ± 1.00	1.75 ± 1.24	*↑ (0.0389)	–	–
	*ATP6VOB*	1.04 ± 0.29	0.72 ± 0.25	0.54 ± 0.10	*↓ (<0.05)	**↓ (0.0035)	–
	*FAM82A2*	1.02 ± 0.22	1.05 ± 0.09	1.09 ± 0.09	–	–	–
	*SLC6A6*	1.02 ± 0.19	1.37 ± 0.27	1.39 ± 0.36	*↑ (0.0154)	–	–
	*SLC25A3*	1.05 ± 0.35	1.09 ± 0.43	1.01 ± 0.19	–	–	–
	*TOMM40*	1.05 ± 0.38	0.88 ± 0.30	0.56 ± 0.15	–	*↓ (0.0486)	–
	*ZNF642*	1.02 ± 0.22	0.94 ± 0.32	1.03 ± 0.04	–	–	–

Similar trends are found in DLB and rpDLB when compared with MA cases. Major changes are represented with the corresponding *p*-value, and mRNA levels are expressed as mean fold change ± SD determined by real-time quantitative PCR (RT-qPCR) and analyzed with the ΔΔthreshold cycle (CT) method: *p < 0.05, **p < 0.01, and ***p < 0.001.

### Protein Expression of Mitochondrial Subunits of the Respiratory Chain in MA, DLB, and rpDLB

**Figure 1:**
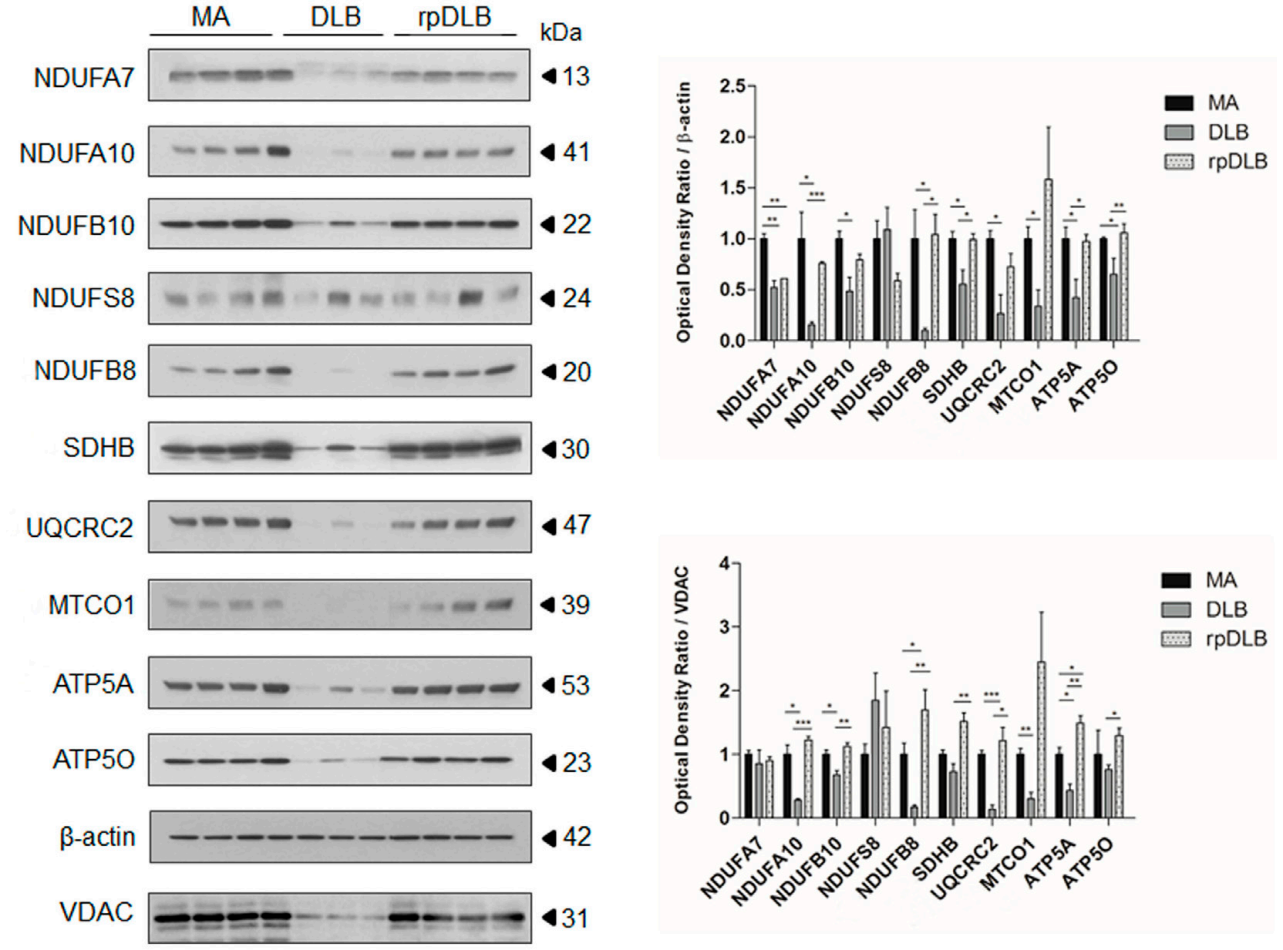
**Protein expression in middle-aged (MA) (*n* = 12), dementia with Lewy bodies (DLB) (*n* = 9) and rapid DLB (rpDLB) (*n* = 4) of subunits of mitochondrial complexes I (NDUFFA7, NDUFA10, NDUFB10, NDUFS8, NDUFB8), II (SDHB), III (UQCRC2), IV (MTCO1), and V (ATP5A, ATP50) normalized with both β-actin- and voltage-dependent anion channel (VDAC)**. Three representative cases are shown in western blots. Diagrams show quantitative values of all assessed cases. Significant decrease in the expression levels of the majority of these subunits is seen in the frontal cortex in DLB and less markedly in rpDLB when compared with MA cases when normalized with β-actin and in most cases with VDAC. Decreased expression of VDAC in DLB probably reflects decrease in the number of mitochondria: *p < 0.05, **p < 0.01, and ***p < 0.001.

Western blots showed marked alterations in DLB when compared with MA cases. The expression levels of complex I subunits NDUFA7, NDUFA10, NDUFB10, and NDUFB8 were significantly reduced (*p* ranging from <0.05 to <0.001) when compared with MA cases and using β-actin for protein normalization, expression of which was maintained in DLB and rpDLB when compared with MA cases. NDUFS8 protein expression was not modified in DLB when compared with MA. Protein levels of SDHB (complex II), UQCRC2 (complex III), MTCO1 (complex IV), and ATP5A and ATP50 (complex V) were also significantly decreased in DLB when compared with MA (*p* < 0.05) using β-actin for protein loading normalization (Figure 1). In contrast, only NDUFA7 was significantly reduced in rpDLB when compared with MA (*p* < 0.01). However, the expression of several subunits showed a trend toward decrease in rpDLB; therefore, significant differences between DLB and rpDLB were restricted to NDUFB8, SDHB, ATP5A, and ATP50 (*p* < 0.01) (Figure 1).

Voltage dependent anion channel (VDAC) expression was reduced in DLB and less markedly in rpDLB when compared with MA thus suggesting a reduced number of mitochondria or reduced mitochondria size. Nevertheless, NDUFA10, NDUFB10, NDUFB8, UQCRC2, MTCO1, and AT5A protein expression was also reduced in DLB cases when using VDAC for normalization (*p* ranging from <0.05 to <0.001). NDUFA7, NDUFS8, and ATP50 levels were similar in DLB and MA cases using VDAC for normalization. Curiously, expression levels of all the assessed subunits were preserved in rpDLB when normalized with VDAC. As a result, levels of NDUFA10, NDUFB10, NDUFB8, SDHB, UQCRC2, ATP5A, and ATP50 were significantly higher in rpDLB when compared with DLB (*p* values ranging from <0.05 to <0.001) (Figure 1).

### Activity of Mitochondrial Complexes I, II, III, IV, and V in Frontal Cortex Area 8 in DLB and rpDLB Cases

**Figure 2:**
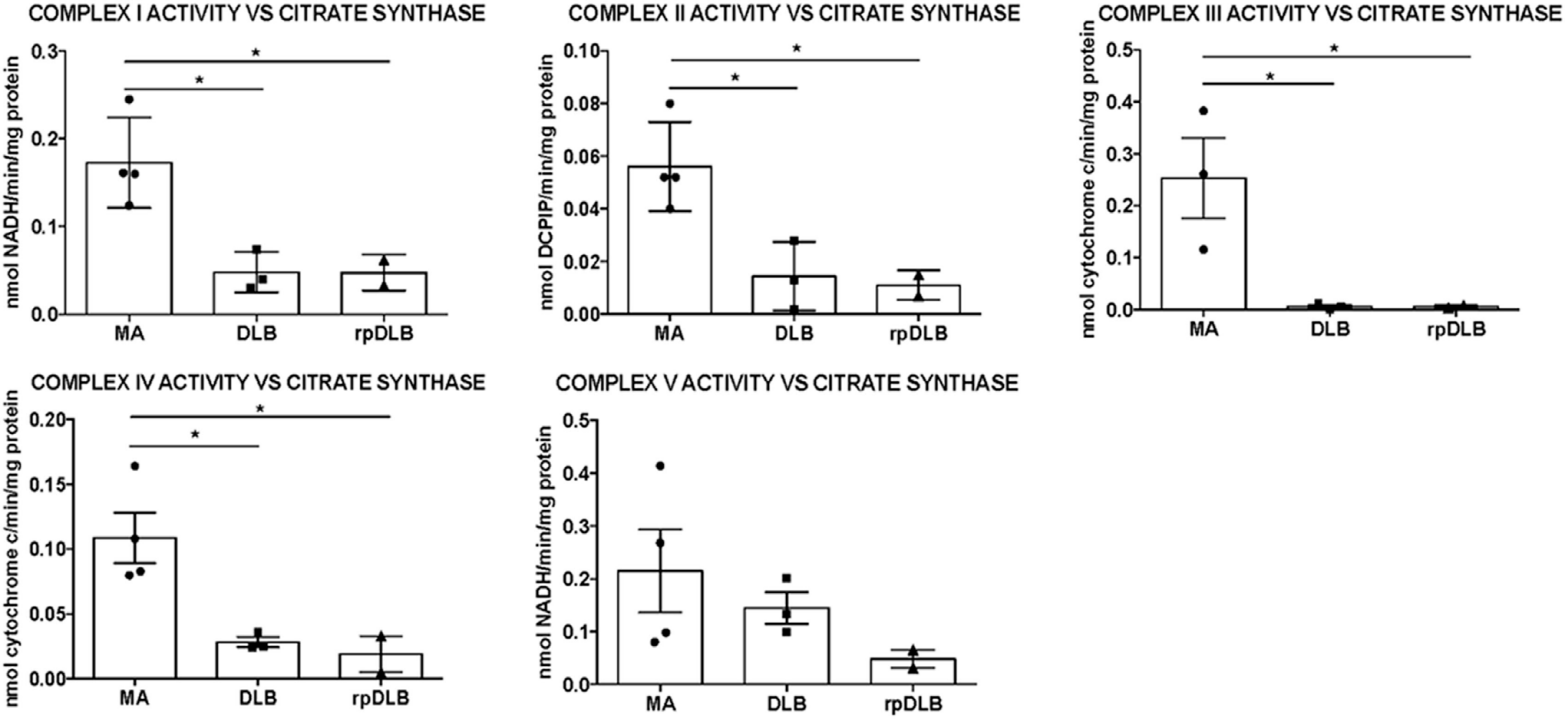
**Mitochondrial enzymatic activities in complex I, II, III, IV, and V in middle-aged (MA), dementia with Lewy bodies (DLB), and rapid DLB (rpDLB)**. All the mitochondrial activities are corrected with the appropriate values of citrate synthase for each sample. Significant decreased activity of complex I, II, III, and IV is observed in DLB and rpDLB when compared with MA. Complex V activity showed a trend toward reduction in DLB and rpDLB: *p < 0.05, **p < 0.01, ***p < 0.001.

Significant decrease in the activity of complexes I, II, III, and IV was detected in frontal cortex area 8 in DLB and rpDLB when compared with MA cases and normalized with the ratio of citrate synthase activity (*p* < 0.05) (Figure 2). No differences were observed when comparing DLB and rpDLB cases. The activity of complex V showed a trend toward decrease in DLB and more clearly in rpDLB but without statistical significance (Figure 2).

### mRNA Expression of Genes Involved in Purine Metabolism in MA, DLB, and rpDLB

The expression of all the assessed genes involved in purine metabolism was altered in the same direction in DLB and rpDLB when compared with MA cases. Changes were greater in rpDLB than in DLB. *ADA, AK1, ENTPD1, NME6*, and *PNP* (*p* < 0.05); *NME1, NME3, NME4* (all of them with *p* < 0.01) and *PRUNE* (*p* < 0.001) were upregulated in rpDLB when compared with MA cases (Table 5). Finally, three genes, *ENTPD2, NME3*, and *PRUNE* were significantly upregulated in rpDLB when compared with DLB (*p* values varied from <0.05 to <0.001) (Table 5).

**Table 5:** **mRNA expression of genes encoding proteins linked to purine metabolism in MA (*n* = 12), DLB (*n* = 9), and rpDLB (*n* = 4)**.

Probes	MA	DLB	rpDLB	MA vs DLB	MA vs rpDLB	DLB vs rpDLB
*ADA*	1.12 ± 0.55	2.09 ± 1.41	2.83 ± 0.57	–	*↑ (0.0123)	–
*AK1*	1.03 ± 0.27	1.13 ± 0.53	1.64 ± 0.17	–	*↑ (0.0321)	–
*AK2*	1.06 ± 0.35	1.26 ± 0.62	1.72 ± 0.35	–	–	–
*AK4*	1.06 ± 0.35	1.96 ± 1.52	2.22 ± 0.73	–	–	–
*AK5*	1.09 ± 0.49	0.83 ± 0.37	1.27 ± 0.53	–	–	–
*AK7*	1.08 ± 0.48	1.05 ± 0.31	1.29 ± 0.31	–	–	–
*APRT*	1.04 ± 0.31	0.79 ± 0.25	0.86 ± 0.28	–	–	–
*DGUOK*	1.03 ± 0.26	0.90 ± 0.32	1.31 ± 0.23	–	–	–
*ENTPD1*	1.09 ± 0.45	1.55 ± 1.04	2.50 ± 0.09	–	*↑ (0.0198)	–
*ENTPD2*	1.01 ± 0.17	0.60 ± 0.19	1.37 ± 0.94	–	–	*↑ (0.0204)
*ENTPD3*	1.07 ± 0.42	0.83 ± 0.27	0.92 ± 0.30	–	–	–
*NME1*	1.05 ± 0.35	1.66 ± 0.95	2.79 ± 1.11	–	**↑ (0.0031)	–
*NME3*	1.04 ± 0.29	1.07 ± 0.38	1.89 ± 0.49	–	**↑ (<0.01)	**↑ (<0.01)
*NME4*	1.01 ± 0.18	1.68 ± 1.03	2.55 ± 0.84	–	**↑ (0.0033)	–
*NME5*	1.05 ± 0.36	0.71 ± 0.41	1.15 ± 0.19	–	–	–
*NME6*	1.03 ± 0.24	1.31 ± 0.19	1.49 ± 0.33	*↑ (<0.05)	*↑ (<0.05)	–
*NME7*	1.05 ± 0.34	0.82 ± 0.40	1.08 ± 0.32	–	–	–
*NT5C*	1.04 ± 0.30	0.94 ± 0.46	1.24 ± 0.36	–	–	–
*NT5E*	1.05 ± 0.34	0.82 ± 0.40	1.08 ± 0.32	–	–	–
*PNP*	1.16 ± 0.74	1.94 ± 0.82	2.91 ± 1.72	–	*↑ (0.0157)	–
*POLR3B*	1.04 ± 0.29	1.03 ± 0.29	0.98 ± 0.19	–	–	–
*PRUNE*	1.01 ± 0.14	1.18 ± 0.26	1.62 ± 0.44	–	***↑ (0.0009)	*↑ (<0.05)

Important variations were detected in rpDLB cases when compared with MA. Changes are represented with the corresponding p-value and mRNA levels are expressed as mean fold change ± SD determined by RT-qPCR and analyzed with the ΔΔCT method: *p < 0.05, **p < 0.01, and ***p < 0.001.

### mRNA Expression Levels of Genes Encoding Nucleolar Proteins and Ribosomal Proteins, and rRNAs18S and 28S in MA, DLB, and rpDLB

Nucleoplasmin 1 (*NPM1*) mRNA was significantly decreased in DLB when compared with MA (*p* < 0.05) (Table 6).

**Table 6:** **mRNA expression levels of genes encoding nucleolar proteins, ribosomal proteins, and 18S and 28S rRNAs in MA (*n* = 12), DLB (*n* = 9), and rpDLB (*n* = 4)**.

	Probes	MA	DLB	rpDLB	MA vs DLB	MA vs rpDLB	DLB vs rpDLB
**Nucleolar proteins**							
	*NPM1*	1.07 ± 0.45	0.60 ± 0.11	0.71 ± 0.24	*↓ (0.0246)	–	–
	*NCL*	1.075 ± 0.446	0.70 ± 0.17	0.78 ± 0.27	–	–	–
	*UBTF*	1.07 ± 0.43	0.72 ± 0.17	0.83 ± 0.40	–	–	–
**rRNA**							
	*rRNA18S*	1.04 ± 0.28	1.22 ± 0.41	2.12 ± 0.66	–	**↑ (0.0015)	**↑ (<0.01)
	*rRNA28S*	1.12 ± 0.62	1.01 ± 0.25	1.34 ± 1.12	–	–	–
**Ribosomal proteins**							
Large subunit	*RPL5*	1.10 ± 0.14	0.99 ± 0.16	1.23 ± 0.25	–	–	–
	*RPL7*	1.01 ± 0.12	1.19 ± 0.15	1.38 ± 0.20	*↑ (<0.05)	**↑ (0.0014)	–
	*RPL21*	1.03 ± 0.29	2.10 ± 0.65	2.72 ± 0.77	**↑ (<0.01)	***↑ (<0.0001)	–
	*RPL22*	1.01 ± 0.17	0.78 ± 0.18	0.83 ± 0.20	*↓ (0.0305)	–	–
	*RPL23A*	1.04 ± 0.31	1.41 ± 0.28	1.71 ± 0.46	–	*↑ (<0.05)	–
	*RPL26*	1.02 ± 0.21	0.76 ± 0.34	0.71 ± 0.22	–	–	–
	*RPL27*	1.01 ± 0.15	1.04 ± 0.11	1.23 ± 0.34	–	–	–
	*RPL30*	1.03 ± 0.26	1.55 ± 0.34	2.05 ± 0.31	**↑ (<0.01)	*** ↑ (<0.0001)	–
	*RPL31*	1.01 ± 0.15	1.21 ± 0.25	1.55 ± 0.29	–	**↑ (0.0026)	–
Small subunit	*RPS3A*	1.03 ± 0.25	1.36 ± 0.40	1.26 ± 0.22	–	–	–
	*RPS5*	1.01 ± 0.11	1.20 ± 0.17	1.31 ± 0.19	*↑ (<0.05)	*↑ (<0.05)	–
	*RPS6*	1.01 ± 0.17	1.14 ± 0.10	1.70 ± 0.40	–	***↑ (0.0002)	**↑ (<0.01)
	*RPS10*	1.02 ± 0.23	1.04 ± 0.19	1.17 ± 0.30	–	–	–
	*RPS13*	1.01 ± 0.16	1.04 ± 0.11	1.35 ± 0.30	–	*↑ (<0.05)	*↑ (<0.05)
	*RPS16*	1.04 ± 0.29	0.75 ± 0.29	0.52 ± 0.01	–	*↓ (0.0166)	–
	*RPS17*	1.01 ± 0.11	0.90 ± 0.06	1.10 ± 0.28	–	–	–
	*RPS20*	1.01 ± 0.13	0.92 ± 0.14	0.96 ± 0.20	–	–	–

Changes are represented with the corresponding p-value and mRNA levels are expressed as mean fold change ± SD determined by RT-qPCR and analyzed with the ΔΔCT method: *p < 0.05, **p < 0.01, and ***p < 0.001.

*rRNA 18S* expression was increased in rpDLB (*p* < 0.01) when compared with MA and also when compared with DLB (*p* < 0.01) (Table 6).

Genes encoding ribosomal proteins of the large (L) and the small (S) subunits were upregulated in DLB when compared with MA control cases including *RPL7, RPL21, RPL30*, and *RPS5* (*p* values varied from <0.05 to <0.001), but *RPL22* was downregulated in DLB when compared with MA (*p* < 0.05) (Table 6).

*RPL7, RPL21, RPL23A, RPL30, RPL31, RPS5, RPS6*, and *RPS13* were upregulated in rpDLB in comparison to MA (*p* values varied from <0.05 to <0.001); *RPS16* was downregulated (*p* < 0.05) (Table 6). Due to similar trends of gene expression in DLB and rpDLB, only *RPS6* and *RPS13* were significantly upregulated (*p* values ranged from <0.05 to <0.001) in rpDLB when compared with DLB (Table 6).

### Expression of Proteins Involved in Ribosomal Transcription in MA, DLB, and rpDLB

**Figure 3:**
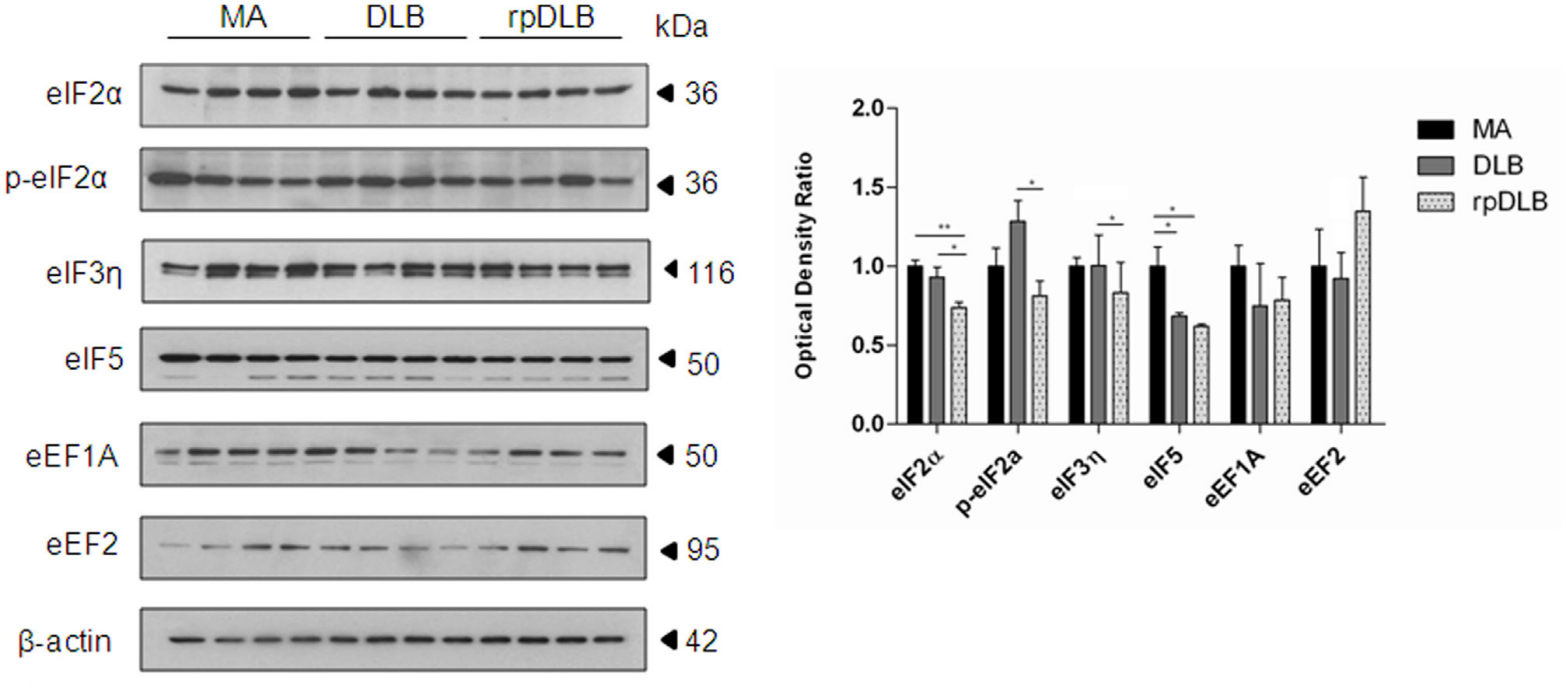
**Protein expression, as revealed by western blotting, of initiation and elongation factors of protein transcription at the ribosome in MA (*n* = 12), dementia with Lewy bodies (DLB) (*n* = 9) and rapid DLB (rpDLB) (*n* = 4) using β-actin for normalization**. Reduced expression of initiation factors is more marked in rpDLB than in DLB, whereas the expression of elongation factors eEF1A and eEF2 is not modified in DLB and rpDLB: *p < 0.05, **p < 0.01, and ***p < 0.001.

Only eIF5 was significantly reduced in DLB when compared with MA cases (*p* < 0.05). However, the expression levels of initiation factors eIF2α and eIF5 were significantly decreased in rpDLB when compared with MA cases (*p* < 0.01 and *p* < 0.05, respectively). eIF2α, p-eIF2α, and eIF3η were significantly decreased (*p* < 0.05) in rpDLB when compared with DLB (Figure 3).

In contrast, no alterations in the expression levels of elongation factors eEF1A and eEF2 were observed in DLB and rpDLB when compared with MA cases (Figure 3).

### mRNA Expression Levels of Cytokines and Mediators of the Innate Inflammatory Response in MA, DLB, and rpDLB

No differences in the expression of twenty-three genes encoding cytokines and mediators of the inflammatory response were detected in DLB when compared with MA. However, significant increase in the levels of *TNF*α (*p* < 0.001) and *CST7* (*p* < 0.05) was found in rpDLB cases when compared with MA (Table 7). *TNF*α and *C1QL1* were significantly upregulated in rpDLB when compared with DLB (*p* < 0.01 and *p* < 0.05, respectively) (Table 7).

**Table 7:** **mRNA expression levels of genes encoding cytokines and mediators of the innate inflammatory response in MA (*n* = 12), DLB (*n* = 9), and rpDLB (*n* = 4)**.

	Probes	MA	DLB	rpDLB	MA vs DLB	MA vs rpDLB	DLB vs rpDLB
**Anti-inflammatory cytokines**							
IL10 family	*IL10*	1.36 ± 0.99	0.70 ± 0.38	1.37 ± 0.97	–	–	–
	*IL10RA*	1.13 ± 0.61	0.84 ± 0.29	1.40 ± 0.66	–	–	–
	*IL10RB*	1.04 ± 0.29	0.85 ± 0.29	1.24 ± 0.60	–	–	–
TGF family	*TGFB1*	1.11 ± 0.50	0.93 ± 0.39	1.40 ± 0.55	–	–	–
	*TGFB2*	1.08 ± 0.46	1.28 ± 0.46	1.40 ± 0.48	–	–	–
**Pro-inflammatory cytokines**							
	*IL6*	1.20 ± 0.78	1.18 ± 0.99	3.25 ± 2.87	–	–	–
	*IL6ST*	1.04 ± 0.31	0.92 ± 0.18	1.33 ± 0.44	–	–	–
	*IL8*	1.12 ± 0.60	1.37 ± 0.83	1.39 ± 0.73	–	–	–
	*IL1*β	1.61 ± 1.57	1.50 ± 1.81	1.22 ± 0.71	–	–	–
TNFα family	*TNF*α	1.14 ± 0.57	1.52 ± 0.51	4.28 ± 1.28	–	***↑ (0.0005)	**↑ (<0.01)
	*TNFRSF1A*	1.08 ± 0.41	1.57 ± 1.06	1.60 ± 0.84	–	–	–
**Inflammation mediators**							
TLRs	*TLR4*	1.06 ± 0.38	1.56 ± 1.11	1.74 ± 0.99	–	–	–
	*TLR7*	1.36 ± 1.04	0.83 ± 0.57	1.21 ± 0.52	–	–	–
Colony-stimulating factors	*CSF1R*	1.15 ± 0.55	0.80 ± 0.43	1.12 ± 0.29	–	–	–
	*CSF3R*	1.24 ± 0.79	1.14 ± 0.65	2.21 ± 0.44	–	–	–
Complement system	*C1QL1*	1.04 ± 0.31	0.95 ± 0.27	1.74 ± 0.99	–	–	*↑ (0.0366)
	*C3AR1*	1.27 ± 0.87	0.97 ± 0.66	2.22 ± 1.61	–	–	–
	*C1QTNF7*	1.15 ± 0.64	0.85 ± 0.37	0.97 ± 0.49	–	–	–
Cathepsins	*CTSC*	1.37 ± 1.25	0.86 ± 0.54	0.92 ± 0.09	–	–	–
	*CTSS*	1.36 ± 1.24	0.91 ± 0.53	1.11 ± 0.46	–	–	–
Integrin family &	*ITGB2*	1.33 ± 0.97	0.98 ± 0.62	1.73 ± 1.04	–	–	–
CTL/CTLD superfamily	*CLEC7A*	1.24 ± 0.76	0.95 ± 0.46	1.11 ± 0.39	–	–	–
	*CST7*	1.44 ± 1.17	2.77 ± 1.89	4.46 ± 1.99	–	*↑ (0.0307)	–

Changes are represented with the corresponding p-value and mRNA levels are expressed as mean fold change ± SD determined by RT-qPCR and analyzed with the ΔΔCT method: *p < 0.05, **p < 0.01, and ***p < 0.001.

### Microglia and TNFα in MA, DLB, and rpDLB

**Figure 4:**
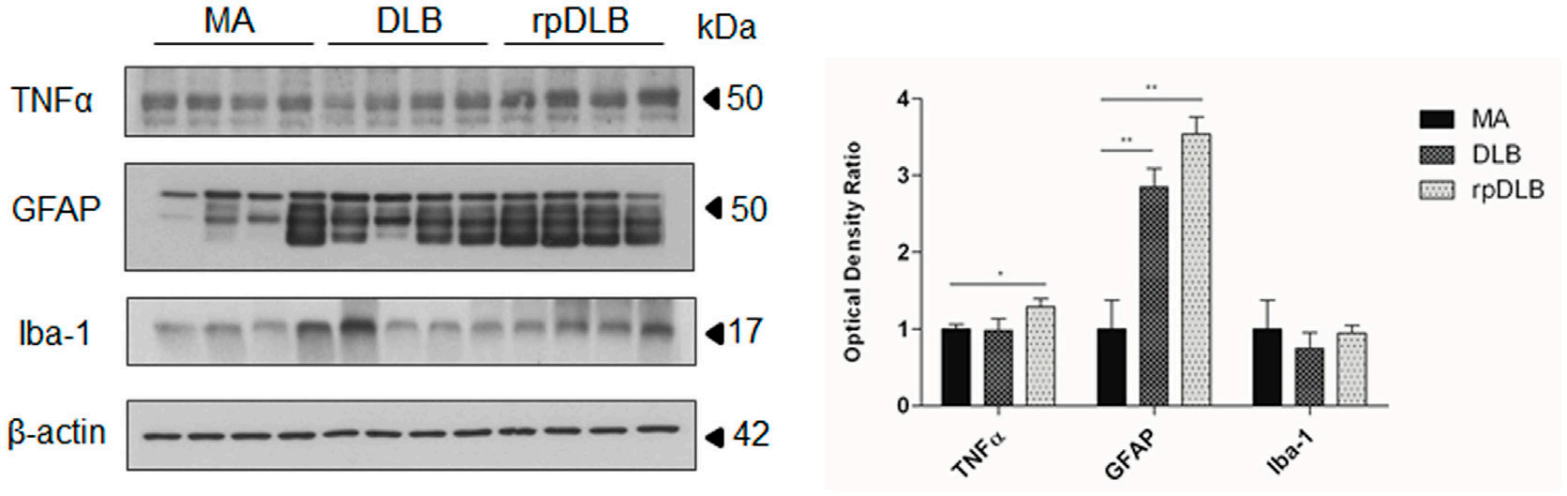
**TNFα, GFAP, and Iba-1 protein levels in middle-aged (MA), dementia with Lewy bodies (DLB), and rapid DLB (rpDLB) as revealed by western blotting using β-actin for normalization**. Significant increased GFAP expression occurs in DLB and rpDLB when compared with MA (*p* < 0.01). A significant increase also occurs in TNFα levels in rpDLB when compared with MA (*p* < 0.05): *p < 0.05, **p < 0.01, and ***p < 0.001.

Western blotting revealed a significant increase in GFAP protein levels in DLB and rpDLB when compared with MA cases (*p* < 0.01), whereas the levels of Iba-1 were maintained when compared with MA cases (Figure 4). TNFα protein levels were also significantly increased in rpDLB when compared with MA (*p* < 0.05) (Figure 4).

### mRNA Expression Levels of Olfactory Receptors (ORs) and Taste Receptors (TASRs) in MA, DLB, and rpDLB

Increased expression of *OR2D2, OR4F4, OR11H1*, and *OR52H1* was observed in DLB when compared with MA cases (*p* < 0.05). Upregulation of *OR2D2* and OR2T33 was significantly higher in rpDLB compared with MA (*p* < 0.05). Significant differences between DLB and rpDLB were restricted to *OR2T33* and *OR4F4* (*p* < 0.001 and *p* < 0.05, respectively) (Table 8).

**Table 8:** **mRNA expression levels of genes encoding olfactory receptors (ORs) and taste receptors (TASRs) in MA (*n* = 12), DLB (*n* = 9), and rpDLB (*n* = 4)**.

Probes	MA	DLB	rpDLB	MA vs DLB	MA vs rpDLB	DLB vs rpDLB
**ORs**						
*OR2D2*	1.30 ± 0.77	3.39 ± 2.57	4.94 ± 0.89	*↑ (<0.05)	**↑ (0.0018)	–
*OR2J3*	1.13 ± 0.54	1.10 ± 0.55	1.55 ± 0.83	–	–	–
*OR2L13*	1.36 ± 0.86	1.22 ± 0.88	1.09 ± 0.92	–	–	–
*OR2T1*	1.09 ± 0.49	1.63 ± 0.97	1.71 ± 0.35	–	–	–
*OR2T33*	1.24 ± 0.83	0.80 ± 0.38	3.60 ± 1.44	–	**↑ (0.01)	***↓ (0.0004)
*OR4F4*	1.20 ± 0.52	2.22 ± 1.09	0.80 ± 0.32	*↑ (<0.05)	–	*↓ (0.05)
*OR6F1*	1.02 ± 0.22	1.63 ± 1.40	2.14 ± 0.86	–	–	–
*OR10G8*	1.11 ± 0.53	1.23 ± 0.44	0.67 ± 0.21	–	–	–
*OR11H1*	1.07 ± 0.37	3.15 ± 2.37	2.26 ± 0.74	*↑ (0.0236)	–	–
*OR51E1*	1.11 ± 0.49	1.94 ± 1.57	1.83 ± 0.96	–	–	–
*OR52H1*	1.18 ± 0.65	3.60 ± 3.00	4.25 ± 1.25	*↑ (0.0256)	–	–
*OR52L1*	1.05 ± 0.35	1.66 ± 1.14	1.06 ± 1.02	–	–	–
*OR52M1*	1.09 ± 0.52	1.10 ± 0.87	1.84 ± 0.36	–	–	–
**TASR**						
*TAS2R4*	1.03 ± 0.27	1.82 ± 0.78	2.16 ± 0.78	*↑ (<0.05)	**↑ (0.0029)	–
*TAS2R5*	1.03 ± 0.25	2.25 ± 1.03	2.12 ± 0.33	**↑ (0.0013)	*↑ (<0.05)	–
*TAS2R10*	1.13 ± 0.50	1.68 ± 0.64	1.88 ± 0.44	–	–	–
*TAS2R13*	1.11 ± 0.49	1.63 ± 0.62	1.99 ± 0.06	–	*↑ (0.0201)	–
*TAS2R14*	1.15 ± 0.57	2.51 ± 1.59	4.14 ± 1.16	*↑ (<0.05)	***↑ (0.0004)	–
*TAS2R50*	1.08 ± 0.40	1.54 ± 1.15	2.34 ± 1.16	–	–	–

Changes are represented with the corresponding p-value, and mRNA levels are expressed as mean fold change ± SD determined by RT-qPCR and analyzed with the ΔΔCT method: *p < 0.05, **p < 0.01, and ***p < 0.001.

*TAS2R4, TAS2R5*, and *TAS2R14* were upregulated in DLB when compared with MA (*p* values varied from <0.05 to <0.01). *TAS2R4, TAS2R5, TAS2R13*, and *TAS2R14* expression was significantly increased in rpDLB in comparison to MA cases (*p* values from <0.05 to <0.001) (Table 8).

### Soluble Aβ40 and Aβ42, Membrane-Associated β-Amyloid, Tau Oligomeric Species, and α-Synuclein Oligomeric Species in Total Homogenate Fractions

**Figure 5:**
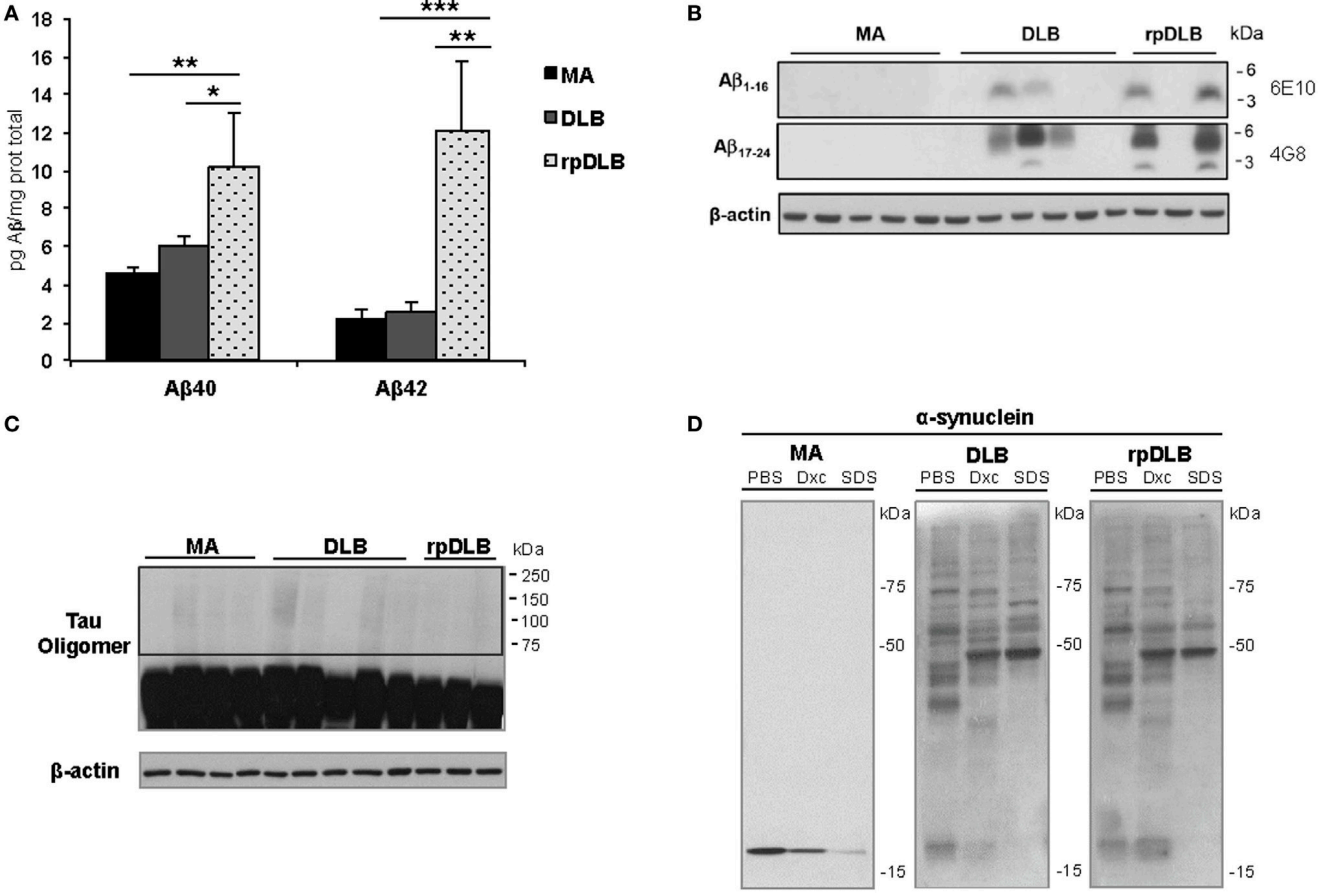
**Soluble Aβ40 and Aβ42, membrane-associated β-amyloid, tau oligomers, and α-synuclein oligomeric species in total homogenate fractions in middle-aged (MA) (*n* = 10), dementia with Lewy bodies (DLB) (*n* = 10) and rapid DLB (rpDLB) (*n* = 4)**. **(A)** Soluble Aβ40 and Aβ42 levels are similar in MA and DLB cases but soluble Aβ40 and Aβ42 are significantly increased in rpDLB when compared with MA and DLB; **(B)** membrane-associated β-amyloid is detected in DLB and rpDLB but not in MA cases as revealed with human amyloid-beta protein antibodies 4G8 and 6E10; **(C)** no tau oligomers are detected in DLB and rpDLB even after membrane over-exposition; **(D)** α-synuclein oligomers are present equally in DLB and rpDLB, which is in contrast with the lack of α-synuclein oligomers in MA. The figures are representative of four MA, four DLB, and four rpDLB; statistical values represent the totality of samples: *p < 0.05, **p < 0.01, and ***p < 0.001.

In spite of there being no apparent differences in β-amyloid plaque burden in DLB and rpDLB, significant increases in soluble Aβ40 and Aβ42 levels were seen in rpDLB when compared with DLB (*p* < 0.05 and *p* < 0.01, respectively) (Figure 5A). Curiously, levels in DLB were similar to those in controls. However, no differences between DLB and rpDLB were seen regarding β-amyloid associated with membranes (Figure 5B), which in AD has a close correlation with fibrillar β-amyloid in amyloid plaques (78).

Regarding tau pathology, western blots of total homogenates disclosed no tau oligomers in frontal cortex in DLB and rpDLB (Figure 5C) thus being in accordance with the small quantity of NFT in the frontal cortex in the present series.

Finally, α-synuclein oligomers were observed in DLB and rpDLB in contrast with the lack of α-synuclein oligomers in MA cases. Importantly, the band pattern and the density of the bands of oligomers were similar in DLB and rpDLB (Figure 5D).

## Discussion

The present study was undertaken to identify alterations of several metabolic pathways, which may participate in age-related DLB pathology including mitochondrial function and energy metabolism, purine metabolism, protein synthesis machinery, inflammation and certain recently discovered new ectopic ORs and TASRs expressed in brain.

The focus of the study was to learn about biochemical alterations beyond the well-known modifications of target proteins in DLB such as α-synuclein and β-amyloid. Present biochemical studies have shown similar percentages of Aβ40 and Aβ42 in plaques in DLB and rpDLB abnormal solubility and aggregation of α-synuclein and increased β-amyloid bound to membranes in the frontal cortex in DLB and rpDLB. In contrast, no differences in tau oligomers were found between MA and DLB cases. Whether levels of soluble Aβ40 and Aβ42 are within control values in DLB in spite of the presence of plaques (73, 74) needs further study.

### Mitochondria and Energy Metabolism

Mitochondrial alterations in the frontal cortex are prominent in DLB. *ATP5G2* and *ATP5H* expression is decreased in DLB when compared with MA individuals; protein expression of NDUFA7, NDUFA10, NDUFB8, SDHB, UQCRC2, MTCO1, ATP5A, and ATP50 is reduced. No differences in expression are found in rpDLB in contrast to DLB. NDUFA7 protein levels are significantly reduced in rpDLB and significant differences between DLB and rpDLB are restricted to NDUFB8, SDHB, ATP5A, and ATP50. It is worth stressing that these alterations are not the mere consequence of mitochondrial loss in DLB. Expression levels of VDAC are decreased in DLB but the expression levels of the mentioned subunits are reduced even considering VDAC for normalization of protein levels. It can be argued that the rapid course of the disease results in a reduced harmful impact on mitochondria in rpDLB when compared with DLB. Despite differences in gene and protein expression, mitochondrial enzymatic activity of complexes I, II, III, and IV is significantly decreased in frontal cortex area 8 in DLB and rpDLB. Therefore, the present observations point to altered mitochondrial function in frontal cortex as a major factor in the pathogenesis of DLB and rpDLB.

Regarding energy metabolism, *ATP4A* and *SLC6A6* are upregulated in DLB, while *ATP6V0B* is downregulated in DLB and rpDLB. TOMM40 is downregulated in rpDLB *ATP4A* encodes a membrane-bound P-type ATPase, which permits ion transport through cell membranes (79). *SLC6A6* encodes a taurine transporter (SLc6a6/TauT) involved in the uptake of gamma-aminobutyric acid (GABA) (80). Whether these changes have implications in GABA metabolism in DLB is not known although GABA levels in the CSF in DLB are not altered when compared with normal individuals (81). *ATP6V0B* encodes ATPase H+ transporting Vo subunit b, which is involved in protein sorting, zymogen activation, receptor-mediated endocytosis, and synaptic vesicle proton gradient generation (82). *TOMM40* encodes a translocase of outer mitochondrial membrane which is required for protein transfer into mitochondria (83). Together, these results suggest energy metabolism impairment in frontal cortex in DLB and rpDLB.

### Purine Metabolism

Several genes encoding enzymes linked to purine metabolism are upregulated in DLB and rpDLB although significant values are only obtained when comparing rpDLB with MA cases. These include *ADA, AK1, ENTPD1, NME1, NME3, NME4, NME6, PNP*, and *PRUNE*. However, only three genes, *ENTPD2, NME3*, and *PRUNE*, are significantly upregulated in rpDLB when compared with DLB.

Purines and pyrimidines are the core of DNA, RNA, nucleosides, and nucleotides. Nucleotides are involved in cell signaling and energy metabolism, and purine bases are also cofactors of several enzymatic reactions (84–87). Adenylate kinases participate in the phosphorylation of AMP to ADP and dAMP to dATP (88, 89). The *NME* gene family encodes nucleotide diphosphate kinases, which are involved in the phosphorylation of nucleotide diphosphates to form nucleotide triphosphates. These enzymes are enriched in synapses. Increased *NME* mRNA expression in DLB contrasts with its decrease in AD (90, 91) and suggests a compensatory role of *NME* in response to reduced synapses in DLB. The product of *PRUNE* participates in the metabolism of guanosine pentaphosphate and tetraphosphate and is linked to *NME* in memory conservation (92). Deoxyguanosine kinase (encoded by *DGUOK*) phosphorylates purine deoxyribonucleosides in the mitochondrial matrix (93–95). Adenine phosphoribosyltransferase is related to adenine metabolism and catalyses the phosphorylation reaction, whereas ADA de-aminates adenosine.

Furthermore, ectonucleoside triphosphate diphosphohydrolases, encoded by *ENTPD* genes, hydrolyze the terminal phosphate group of nucleoside tri- and diphosphates to form di- and monophosphates, thus controlling extracellular ATP concentrations (96), adenosine-activated type I receptors, nucleotide-activated type 2 ligand-gated ion channels, and metabotropic P2Y receptors (97). Finally, ecto 5′-nucleotidase catalyzes the generation of adenosine from degradation of AMP in the extracellular space. In brain, ectonucleotidases are involved in several functions including modulation of synaptic transmission, ATP-mediated propagation of calcium waves in glial cells, neurogenesis, microglial function, and blood flow (98).

Present findings show marked alterations in the expression of enzymes involved in purine metabolism in the frontal cortex in DLB and rpDLB.

### Protein Synthesis

Decreased expression of NPM1 is found in the frontal cortex in DLB, which may be indicative of nucleolar stress and linked to altered ribosomal biogenesis (99–103).

Alterations in the expression of genes encoding ribosomal proteins are seen in DLB and rpDLB, including up- and downregulation of RPLs, thus suggesting impaired ribosome biogenesis (104–107). This is accompanied by reduced protein expression of several initiation factors of transcription at the ribosome, which are more marked in rpDLB than in DLB. However, the expression levels of elongation factors eEF1A and eEF2 are preserved in DLB and rpDLB. Although the direct study of protein synthesis is not possible in human postmortem samples due to postmortem delay between death and tissue processing, the present findings indicate that the machinery of protein synthesis is altered in DLB (108–110). Importantly, altered expression of proteins involved in transcription at the ribosome is more severe in rpDLB than in DLB.

### Inflammatory Responses

No significant differences in gene expression of several cytokines and mediators of the inflammatory response are seen in DLB. These data are in line with the observation of no major increase in Iba1 protein levels and CD68 expression in frontal cortex in DLB when compared with MA individuals. They are also in agreement with previous observations showing very limited activation of microglia in DLB (111, 112).

Subtle change refers to *TNF*α gene expression in rpDLB. Whereas *TNF*α mRNA is not altered in DLB, significant *TNF*α mRNA upregulation and increased TNFα protein levels occur in the frontal cortex in rpDLB. TNFα is involved in several metabolic pathways, particularly facilitating gene transcription, activating of the JNK pathway and promoting apoptosis *via* caspase-dependent and caspase-independent signaling (113). Therefore, TNFα upregulation in rpDLB may have functional implications. Further molecular studies are needed to elucidate activated molecules of the TNFα pathways in rpDLB.

### Olfactory and Taste Brain Receptors

Olfactory and taste receptors are widely expressed in human and rodent brain including the cerebral cortex. ORs and TASRs in brain are accompanied by all downstream molecules that permit a functional signaling pathway, and they are functional, as revealed in culture neurons under appropriate stimuli (114, 115).

The expression of several genes encoding ORs is increased in DLB and rpDLB. Three out of six TASR are upregulatred in DLB and four genes in rpDLB.

The function of ORs and TASRs in brain is not known although it has been postulated that they may participate in intra- and extracellular signaling in association, or not, with other receptors. Identification of natural ligands of brain ORs and TASRs should yield insights about the function of these ectopic receptors (114).

### Mitochondrial Function, Inflammation, and Deregulation of ORs and TASRs in Frontal Cortex Discriminate DLB and AD

The present study reveals disease-specific alterations when comparing the present results in DLB with available data for AD in the same region, the frontal cortex, at similar stages of disease progression.

Mitochondrial alterations and impaired activity of complex V are early events in the entorhinal cortex in AD (116). *NDUFA2, NDUFB3, UQCR11, COX7C, ATPD, ATP5L*, and *ATP50* gene expression is reduced in the entorhinal cortex with disease progression, and this is accompanied by impaired activity of complexes I, II, and V (72). In contrast, RNA expression levels of several subunits of complexes I–V and enzymatic activities of complex I, II, IV, and V are preserved in frontal cortex in AD even at stages V–VI of Braak in individuals with mean ages similar to those of assessed DLB cases (72). Therefore, mitochondrial dysfunction in frontal cortex markedly differs in AD and DLB, with detrimental effects in most mitochondrial complexes in DLB in comparison with AD.

There is a large body of information demonstrating microglial responses and increased expression of inflammatory markers in the cerebral cortex in AD (73, 74, 117–131). In contrast, inflammatory responses are very limited in DLB. Therefore, inflammation is characteristically an important factor in AD pathogenesis, whereas it has low impact in DLB.

Regarding purine metabolism, *ENTPD2, NME3, PNP*, and *PRUNE* RNAs are deregulated in the frontal cortex in AD (91), whereas *ADA, AK1, ENTPD1, NME1, NME3, NME4, NME6, PNP*, and *PRUNE* are upregulated in rpDLB with changes less marked in DLB.

Severe alterations of the machinery involved in protein synthesis from the nucleolus to the ribosome have been observed in the entorhinal cortex in AD (132), but no similar data are available in AD frontal cortex.

Finally, *OR4F4* mRNA expression levels are increased in frontal cortex area 8 at stages III–IV, and *OR52L1* mRNA at Braak stages III–IV and V–VI in AD (133). Regarding TASRs, no modification in the mRNA expression levels is observed in frontal cortex area 8 at any stage of AD (133). These observations in AD are in contrast with the extensive deregulation of ORs and TASRs in the frontal cortex in DLB, thus indicating marked differences in the regulation of these brain receptors in DLB when compared with AD.

### Specific Traits in rpDLB Compared with DLB

No neuropathological differences are seen between DLB and rpDLB in accordance with other studies (58). Moreover, rpDLB has similar biochemical profiles regarding mitochondrial gene and protein expression, protein synthesis, and expression of ORs and TASRs. However, gene and protein expression of subunits of mitochondrial respiratory complexes are more pronounced in DLB when compared with rpDLB, whereas alterations in purine metabolism and initiation of protein translation are more pronounced in rpDLB when compared with DLB. Due to the small number of rpDLB cases available for biochemical studies, these observations need further validation.

Two additional traits which differentiate rpDLB and DLB merit a comment. The first of these is the higher levels of soluble Aβ40 and Aβ42 in rpDLB in spite of similar plaque burden and β-amyloid associated with membranes in rpDLB when compared with DLB. In this line, modified characteristics of Aβ42 have been described in rapidly progressive AD (134), thus suggesting that β-amyloid conformers pace disease progression in AD. It can be suggested that higher levels of soluble β-amyloid oligomeric species, known to be toxic to nerve cells (135–140), can precipitate disease progression in rpDLB. The second differential aspect is the higher levels of TNFα in rpDLB when compared with DLB, which can also be toxic to nerve cells.

## Conclusion

Molecular alterations in the cerebral cortex in DLB include (i) deregulated expression of several mRNAs and proteins of mitochondrial subunits and reduced activity of complexes I, II, III, and IV of the mitochondrial respiratory chain; (ii) reduced expression of selected molecules involved in energy metabolism and increased expression of enzymes involved in purine metabolism; (iii) abnormal expression of certain nucleolar proteins, rRNA18S, genes encoding ribosomal proteins and initiation factors of the transcription at the ribosome; (iv) discrete inflammation; and (v) marked deregulation of brain ORs and TASRs. Severe mitochondrial dysfunction involving activity of four complexes, minimal inflammatory responses, and dramatic altered expression of ORs and TASRs discriminate DLB from AD. Altered solubility and aggregation of α-synuclein, increased β-amyloid bound to membranes, and absence of soluble tau oligomers are common in DLB and DLB with rapid progression. Increased soluble β-amyloid 1–40 and β-amyloid 1–42, and increased TNFα mRNA and protein expression, distinguish DLB with rapid progression from typical DLB. Since high levels of soluble β-amyloid species are toxic for nerve cells and TNF-α.

## Author Contributions

PG-E carried out RT-qPCR, western blots, and enzymatic activities. IL-G performed the studies of beta-amyliid and tau oligomers. OG-R participated in the selection of cases and in the neuropathological study. MG-G and AK helped PG-E. FL and SZ helped in molecular studies. MC helped in the processing of samples. JR helped in the design of molecular studies in rapid dementias. IZ helped in the design of the study of rapid dementias. EG carried out the neuropathological and supervised the clinical and pathological relations. IF directed the study, supervised the work, wrote the advanced version of the manuscript, which was circulated among the authors for comments and suggestions, and prepared the final version for submission.

## Conflict of Interest Statement

The authors declare that the research was conducted in the absence of any commercial or financial relationships that could be construed as a potential conflict of interest.
