# Supplementary material for: Dementia with Lewy Bodies: Molecular Pathology in the Frontal Cortex in Typical and Rapidly Progressive Forms
Source: Front Neurol. 2017 Mar 13;8:89. doi: 10.3389/fneur.2017.00089 (PMC5346561; doi:10.3389/fneur.2017.00089)

Supplementary Figure I: Representative western blotting of some MA, DLB and rpDLB samples using eukaryotic initiation factor 3 η (eIF3η) and ATP synthase H+ transporting mitochondrial F1 complex, O subunit (ATP5O) antibodies in order to show the whole membrane.

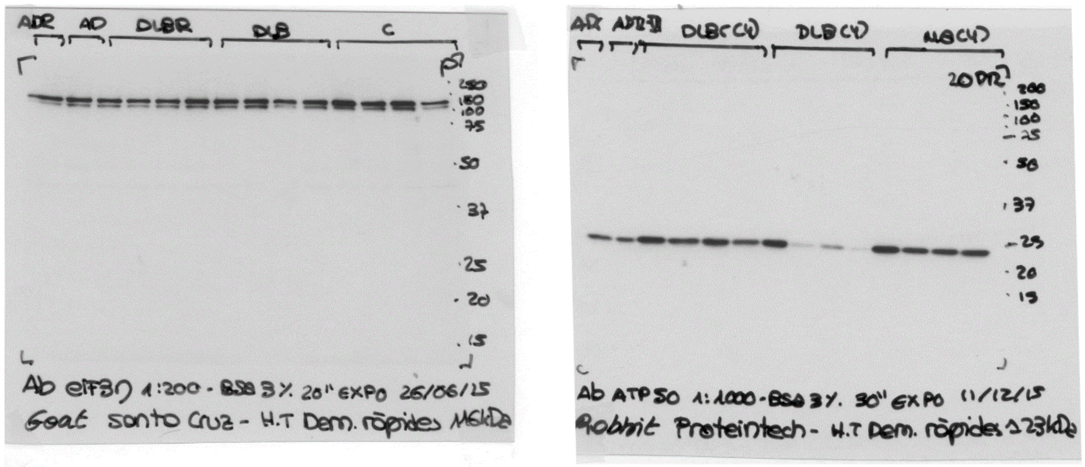

Supplement: Supplementary file 1 [file Image_1.PDF]
