# Supplementary material for: Dementia with Lewy Bodies: Molecular Pathology in the Frontal Cortex in Typical and Rapidly Progressive Forms
Source: Front Neurol. 2017 Mar 13;8:89. doi: 10.3389/fneur.2017.00089 (PMC5346561; doi:10.3389/fneur.2017.00089)
Supplement: Supplementary file 2 [file Table_1.PDF]

Supplementary Table I: Antibodies used for western blotting in the present study.

Antibody	Reference	Supplier	Host	WB
α-synuclein oligomer-specific	AS132718	Agrisera (Vännas, Sweden)	m	1/1,000
Amyloid β				
Tau				
**Initiation and elongation factors**				
anti-eukaryotic translation initiation factor 2 (eIF2-α)	5A5	Thermo Scientific (Waltham, MA, USA)	m	1/50
anti-phospho-eIF2-alpha pSer51 (p-EIF2-α)	S.674.5	Thermo Scientific (Waltham, MA, USA)	rb	1/50
anti-eukaryotic translation initiation factor 3η (eIF3η)	sc-28857	Santa Cruz (Dallas, Texas, USA)	rb	1/200
anti-eukaryotic translation initiation factor 5 (eIF5)	sc-282	Santa Cruz (Dallas, Texas, USA)	rb	1/400
anti-eukaryotic elongation factor 1A (eEF1A)	2551	Cell signaling (Danvers, MA, USA)	rb	1/100
anti-eukaryotic elongation factor 2 (eEF2)	2332	Cell signaling (Danvers, MA, USA)	m	1/1,000
**Inflammatory elements**				
anti-tumor necrosis factor alpha (TNF-α)	ab1793	Abcam (Cambridge, UK)	m	1/500
anti-glial fibrillary acidic protein (GFAP)	Z0334	Dako (Glostrup, Denmark)	rb	1/30,000
anti-Ionized calcium binding adaptor molecule 1 (Iba1)	019-19741	Wako (Osaka, Japan)	rb	1/500
**Mitochondrial components**				
anti-NADH Dehydrogenase (Ubiquinone) 1 Alpha Subcomplex, 7(NDUFA7)	15300-1-AP	Proteintech (Rosemont, USA)	rb	1/100
anti-NADH Dehydrogenase (Ubiquinone) 1 Alpha Subcomplex, 10 (NDUFA10)	GTX114572	Genetex (California, USA)	rb	1/2,500
anti-NADH Dehydrogenase (Ubiquinone) 1 Beta Subcomplex, 10 (NDUFB10)	15589-1-AP	Proteintech (Rosemont, USA)	rb	1/2,400
anti-NADH Dehydrogenase (Ubiquinone) Fe-S Protein 8 (NDUFS8)	GTX114119	Genetex (California, USA)	rb	1/1,000
anti-oxidative phosphorylation (OXPHOS)	MS601	Mitoscience (Eugene, Oregon, USA)	m	1/10,000
anti-ATP Synthase, H+ Transporting, Mitochondrial F1 Complex, O Subunit (ATP5O)	10994-1-AP	Proteintech (Rosemont, USA)	rb	1/1,000
anti-β-actin	A5316	Sigma-Aldrich (St Louis, MO, USA)	m	1/30,000
anti-voltage-dependent anion channel (VDAC)	ab15893	Abcam (Cambridge, UK)	rb	1/500
